# Same hospital, same price, different protection: out-of-pocket expenditure for emergency trauma under public insurance, private insurance and self-payment in India

**DOI:** 10.12688/f1000research.188694.1

**Published:** 2026-10-08

**Authors:** Rajesh Kamath, Amisha Thakar, Ravichandran Nair, Akhila Doddamani, Reena Verma, R. Somasundaram, Anindita Ghosh

**Affiliations:** 1 Department of Healthcare and Hospital Management, Prasanna School of Public Health, Manipal Academy of Higher Education, Manipal, India; 2 Department of Social and Health Innovation, Prasanna School of Public Health, Manipal Academy of Higher Education, Manipal, India; 3 Department of Community Medicine, Kasturba Medical College, Manipal Academy of Higher Education, Manipal, India; 4 School of Nutrition and Dietetics, Symbiosis Skills and Professional University, Pimpri-Chinchwad, Maharashtra, 412101, India; 5 College of Management, SRM Institute of Science and Technology (Deemed to be University) - Tiruchirappalli Campus, Tiruchirappalli, Tamil Nadu, 621105, India

**Keywords:** out-of-pocket expenditure; road traffic accidents; AB-PMJAY; private health insurance; financial risk protection; catastrophic health expenditure; India

## Abstract

**Background:**

Out-of-pocket expenditure (OOPE) finances 43.4% of total health expenditure in India, and emergency trauma—unforeseeable, unpostponable and costly—is among the sharpest tests of whether a health-financing mechanism protects households from catastrophic spending. Insured and uninsured patients are normally treated in different facilities at different tariffs, confounding the effect of the payment mechanism with the setting in which care is given.

**Methods:**

We compared OOPE across three financing arrangements—Ayushman Bharat–Pradhan Mantri Jan Arogya Yojana (AB-PMJAY), private health insurance and self-payment—using billing and admission records for 1,599 of 1,711 consecutive road traffic accident admissions through the emergency department of a 2,000-bed tertiary teaching hospital and regional trauma centre in coastal Karnataka, India, between July 2023 and July 2024. All groups were treated at the same institution under the same price schedule, so total charges are directly comparable, isolating the effect of the payment mechanism.

**Results:**

Of 1,599 patients, 63 (3.9%) were AB-PMJAY beneficiaries, 262 (16.4%) were privately insured and 1,274 (79.7%) were uninsured. Median total charges were statistically indistinguishable across groups (₹90,979, ₹86,170 and ₹85,091; Kruskal–Wallis p = 0.70). Every AB-PMJAY beneficiary incurred zero OOPE. Privately insured patients paid a median of ₹11,598, averaging 25.6% of charges (median 16.7%) and ranging from full reimbursement to none. Uninsured patients bore the entire charge. At a 10%-of-income threshold, catastrophic expenditure affected 0%, 22.5% and 78.7% of the three groups.

**Conclusions:**

Complete protection was achievable and was achieved for every identified beneficiary: the binding failure for the uninsured four-fifths was the absence of cover, not its design. India’s 2025 cashless trauma entitlement addresses that absence, but 23.7% of the uninsured patients here were billed above its ₹1.5 lakh ceiling.

## Introduction

Out-of-pocket expenditure (OOPE) finances 43.4% of total health expenditure in India. The share has fallen from 64.2% a decade earlier, but it remains among the highest in the world, and in absolute terms household spending continues to rise. ^1^ The decline has not been monotonic: the share rose from 39.4% in 2021–22 to 43.4% in 2022–23, a four-percentage-point reversal in a single year. ^1^ Direct payment at the point of care is the dominant mode of health financing, and its consequence is well established: when a household meets a large medical bill from its own pocket, that spending can become catastrophic—conventionally, OOPE exceeding a fixed share of the household’s capacity to pay—and can push families below the poverty line. ^2^ ^,^ ^3^ Financial risk protection, the pooling of health risk so that illness does not translate into impoverishment, is the central objective of Universal Health Coverage and of Sustainable Development Goal target 3.8.2. India’s progress on it remains halting. ^4^

The instrument through which a health system delivers financial protection is the payment mechanism: how a given admission is financed determines how much of its cost the household ultimately bears. India is expanding two pooling mechanisms at once. Ayushman Bharat–Pradhan Mantri Jan Arogya Yojana (AB-PMJAY), the world’s largest publicly funded health assurance scheme, offers cashless cover of up to ₹5 lakh (₹500,000; about US$5,850) per family per year to some 550 million individuals in the poorest two-fifths of the population, and since October 2024 to all citizens aged 70 years and above irrespective of income ^5^; commercial private insurance grows among wealthier households, alongside community-based and mutual-aid schemes that extend voluntary cover to poorer ones. ^6^ Between them, at least 30% of Indians—the ‘missing middle’—hold neither. ^7^ Whether these mechanisms actually protect households, and how completely, is a first-order empirical question for Indian health financing.

Emergency trauma is the sharpest test of that protection. The need for care is unforeseeable, unpostponable and expensive; India recorded 177,175 road-crash deaths and 471,441 recorded injuries in 2024, disproportionately among young, economically productive adults. Crash severity is rising: deaths per 100 crashes climbed from 21.6 in 2005 to 36.3 in 2024, so the financing question is increasingly about the cost of surviving a crash rather than the cost of preventing one. ^8^ ^,^ ^9^ Yet the evidence on how payment mechanisms perform is weak, because comparisons across payer groups are almost always confounded by setting: insured and uninsured patients are drawn from different facilities with different tariffs, case-mix and quality, ^10^ so the protective effect of the mechanism cannot be separated from where and how patients were treated. Injury-related hospitalisation in India consistently shows heavy OOPE, with catastrophic spending affecting roughly one in five injury-affected households and lack of insurance its most consistent determinant. ^11^ ^,^ ^12^ For unplanned emergency admissions the confounding is especially severe: trauma patients, unlike elective patients, cannot choose their hospital.

This study removes that confounding by design. We analysed complete billing records for all road traffic accident admissions over one year at a single tertiary trauma centre where AB-PMJAY beneficiaries, privately insured and uninsured patients are treated by the same clinical teams at the same tariff. Holding the institution and price schedule constant, charges across payer groups can be compared directly; where charges are indistinguishable, differences in OOPE are attributable to the payment mechanism alone. This design extends our earlier finding among 2,715 COVID-19 inpatients at the same institution—where OOPE ran 0% under AB-PMJAY, 21.2% under private insurance and 100% for the uninsured, ^13^ from a pandemic-specific shock to routine emergency trauma, testing whether the protective ordering of financing mechanisms holds across disease contexts. We estimate OOPE among trauma patients, compare financial protection across the three mechanisms, and examine the heterogeneity of protection within private insurance.

## Methods

### Study design and setting

We conducted a retrospective, single-centre analysis of hospital billing and admission records. The study site is a National Accreditation Board for Hospitals and Healthcare Providers (NABH)-accredited tertiary care teaching hospital of approximately 2,000 beds in coastal Karnataka, India, functioning as a major regional trauma referral centre and treating a high volume of emergency cases. Reporting follows the STROBE guideline for cross-sectional studies ^14^; the completed checklist is provided as a supplementary file and each item is addressed in the relevant subsection below.

### Participants and eligibility

The study was scoped to three financing arrangements for emergency trauma care: AB-PMJAY, private health insurance, and self-payment. Patients admitted through the emergency medicine department following a road traffic accident between July 2023 and July 2024 were eligible if their inpatient care was financed through one of these three modes. Of 1,711 road traffic accident admissions in the period, 1,599 met this criterion and were classified into three mutually exclusive groups by payment status at admission: AB-PMJAY beneficiaries, privately insured patients, and uninsured (self-paying) patients. Private health insurance was defined as any non-government, voluntary, premium-financed cover under which a third-party payer settled part of the bill directly with the hospital. The category is heterogeneous: it comprises commercial indemnity products and their third-party administrators together with community-based mutual-aid schemes, the largest of the latter being the Sampoorna Suraksha programme of the Shri Kshethra Dharmasthala Rural Development Project, a subscription-financed scheme covering self-help-group households (n = 65). The remaining patients fell outside the study’s scope: 102 were financed through arrangements outside the three study modes—Employees’ State Insurance (n = 75), the Ex-servicemen Contributory Health Scheme (n = 6), public-sector-undertaking employer schemes (n = 3), a state government scheme (n = 13), a public-sector third-party administrator (n = 3) and free-ward care (n = 2)—whose itemised billing was not extracted and whose financing rules differ from the three modes of interest, and 10 privately insured patients had billing records too incomplete to permit accurate OOPE calculation. The 102 include one record initially segregated into the private insurance group during data preparation that was, on verification of the payer name against the hospital billing system, an employer scheme of a public-sector undertaking: it was reclassified to the other-schemes group before analysis. The analysed set comprised inpatient admissions with complete finance-department billing records. Patients declared dead on arrival, and those who died during emergency-department care before inpatient admission, are not represented in this extract; their medico-legal and administrative billing is handled separately from routine inpatient care.

### Variables and outcome measures

The primary outcome was OOPE, defined as the amount paid by the patient after any insurer reimbursement, expressed both in Indian Rupees (₹) and as a percentage of the total billed amount. For uninsured patients OOPE equalled the total charge; for AB-PMJAY beneficiaries, cashless treatment implied an expected OOPE of zero. Secondary outcomes were the total billed amount, the proportion of patients incurring zero OOPE, catastrophic health expenditure, and insurer-level variation in OOPE among privately insured patients. Amounts were converted to US dollars at ₹85.5 = US$1, the rate prevailing at the time of analysis.

### Data sources

Data were extracted from the hospital’s finance department (itemised billing, insurer-approved amounts and patient payments) and medical records/information-technology systems (admission details and payer identity), and linked at patient level by inpatient number. All records were de-identified before analysis; direct identifiers were removed and access was restricted to the investigators.

### Catastrophic health expenditure

Catastrophic health expenditure (CHE) is conventionally defined as OOPE exceeding a fixed share of a household’s capacity to pay, commonly measured against household consumption expenditure. ^2^ ^,^ ^3^ In the absence of household consumption data, CHE was estimated by comparing each patient’s OOPE against fixed fractions: 10%, 25% and 40% of Karnataka’s per-capita income for 2023–24 (₹332,926, about US$3,894; Economic Survey of Karnataka), used as a standardised capacity-to-pay proxy. ^15^ Results are presented across all three thresholds rather than a single cut-off to convey sensitivity. Because per-capita income is used rather than household consumption, these estimates are indicative of financial risk and are not equivalent to survey-based CHE or impoverishment measures ^2^ ^,^ ^3^; they are interpreted accordingly.

### Statistical analysis

Continuous variables were summarised as medians with interquartile ranges (IQR); billing and OOPE distributions were markedly right-skewed and non-normal on the Shapiro–Wilk test (all p < 0.001), so non-parametric methods were used throughout. Total charges and OOPE were compared across the three groups with the Kruskal–Wallis test, followed by pairwise Mann–Whitney U tests with Bonferroni correction; rank-biserial correlation is reported as an effect size. Ninety-five per cent confidence intervals (CI) for medians were obtained by bias-corrected bootstrap resampling (5000 replications). Categorical variables were compared with Pearson’s χ ^2^ test where no more than 20% of cells had expected counts below five and all expected counts were two or greater; the Fisher–Freeman–Halton exact test was used otherwise. Analyses were performed in Python 3 (pandas, SciPy); two-sided p < 0.05 was considered significant. Road-user category was assigned from the ICD-10 external cause-of-injury code recorded at admission, using the code blocks directly: V01–V09 pedestrian, V10–V19 cyclist, V20–V39 motorcyclist or three-wheeler occupant, V40–V59 car, van or pick-up occupant. Codes for bus and heavy-vehicle occupants, and codes in which the injured person’s role is not specified, were grouped as other or unspecified; no case-note review was undertaken to resolve the latter.

All analyses were performed in Python 3 using the open-source pandas and SciPy libraries. No proprietary software was used.

## Results

### Study population and payer mix

A total of 1,599 RTA patients met the eligibility criteria ( Figure 1). Of these, 63 (3.9%) were covered by AB-PMJAY, 262 (16.4%) by private health insurance, and 1,274 (79.7%) were uninsured ( Table 1). Uninsured patients thus accounted for almost four-fifths of trauma admissions, underscoring the continued predominance of direct payment in emergency trauma financing at this centre.

**Figure 1.**
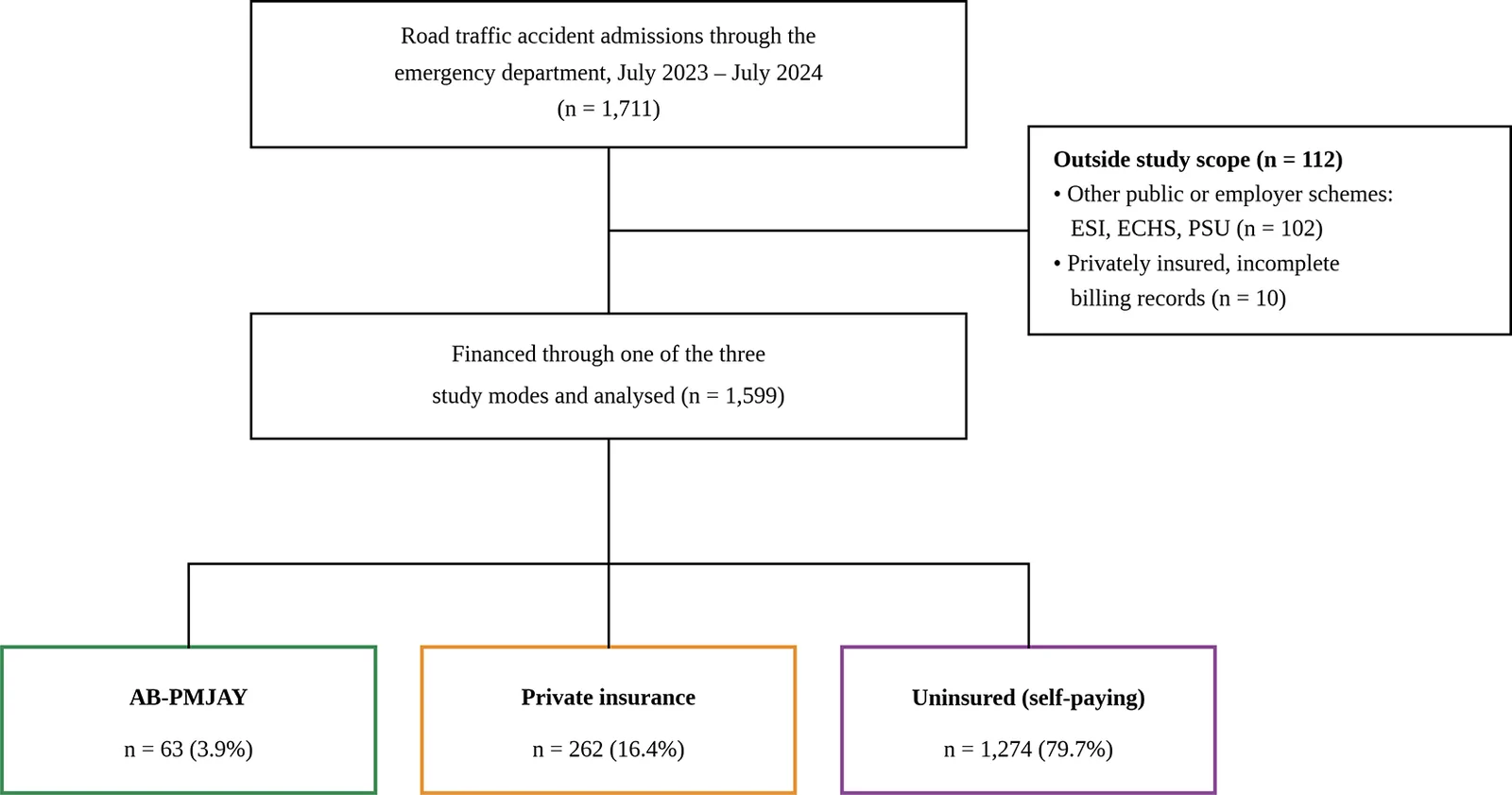
Study flow diagram. ESI, Employees’ State Insurance; ECHS, Ex-servicemen Contributory Health Scheme; PSU, public-sector undertaking.

**Table 1.** Distribution of road traffic accident patients by payer status (n = 1,599).

Payer group	Patients, n	Proportion, %
AB-PMJAY	63	3.9
Private health insurance	262	16.4
Uninsured	1,274	79.7
Total	1,599	100.0

### Age and case-mix differed across payer groups

The three groups were not identical in composition ( Table 2). Uninsured patients were older (median 39 years, IQR 28–52) than AB-PMJAY (34, 24–48) and privately insured patients (34, 23–45), a difference that was statistically significant (Kruskal–Wallis p < 0.001) though modest in magnitude. Case-mix also differed by clinical department (χ ^2^ = 37.4, p < 0.001): orthopaedics was the commonest department in every group, but neurosurgery accounted for almost a third of uninsured admissions (32.8%) and under a quarter of privately insured admissions (23.3%) but only 3.2% under AB-PMJAY. Road-user category also differed across groups (Fisher–Freeman–Halton exact p = 0.007): motorcyclists and three-wheeler occupants were the largest category throughout, accounting for 57.4% of all admissions, but ranged from 47.6% under AB-PMJAY to 62.6% among privately insured patients. Discharge outcomes were favourable in all three groups: 1,554 patients (97.2%) were recorded as improved or recovered, and no in-hospital deaths appear in the analysed set. The distribution of non-favourable outcomes—unchanged, worse, or discharge against medical advice—did differ across payer groups (5, 3 and 37 patients, or 7.9%, 1.1% and 2.9%; Fisher–Freeman–Halton exact p = 0.018), but the counts are small and the gradient does not run against the uninsured. The groups were therefore comparable in what they were billed, and in how they fared clinically, but not identical in their case-mix, a distinction taken up in the discussion.

**Table 2.** Baseline characteristics by payer status: age, clinical department, road-user category and discharge outcome. Other surgical comprises oral, hand, general and plastic surgery; medical/other comprises general medicine, pulmonology, ophthalmology and unclassified units. Road-user category is derived from ICD-10 external-cause-of-injury codes: pedestrian, V01–V09; cyclist, V10–V19; motorcyclist or three-wheeler occupant, V20–V39; car, van or pick-up occupant, V40–V59; other or unspecified comprises bus and heavy-vehicle occupants and all codes in which the injured person’s role is not specified.

Characteristic	AB-PMJAY (n = 63)	Private insurance (n = 262)	Uninsured (n = 1,274)
**Age, years, median (IQR)**	34 (24–48)	34 (23–45)	39 (28–52)
**Clinical department, n (%)**			
Orthopaedics	39 (61.9)	124 (47.3)	526 (41.3)
Neurosurgery	2 (3.2)	61 (23.3)	418 (32.8)
Other surgical	22 (34.9)	64 (24.4)	284 (22.3)
Medical/other	0 (0.0)	13 (5.0)	46 (3.6)
**Road-user category, n (%)**			
Motorcyclist or three-wheeler occupant	30 (47.6)	164 (62.6)	724 (56.8)
Car, van or pick-up occupant	1 (1.6)	10 (3.8)	53 (4.2)
Pedestrian	1 (1.6)	6 (2.3)	23 (1.8)
Cyclist	1 (1.6)	5 (1.9)	2 (0.2)
Other or unspecified	30 (47.6)	77 (29.4)	472 (37.0)
**Discharge outcome, n (%)**			
Improved or recovered	58 (92.1)	259 (98.9)	1,237 (97.1)
Other (unchanged, worse, against advice or diagnosis only)	5 (7.9)	3 (1.1)	37 (2.9)

### Total charges did not differ across payer groups

Median total charges were closely similar across the three groups: ₹90,979 (IQR ₹57,436–₹118,910; 95% CI ₹79,977–₹109,048) for AB-PMJAY, ₹86,170 (IQR ₹40,351–₹144,149) for privately insured, and ₹85,091 (IQR ₹37,666–₹145,825; 95% CI ₹77,649–₹90,289) for uninsured patients. The Kruskal–Wallis test showed no difference in charges across groups (H = 0.72, p = 0.70), and all pairwise comparisons were non-significant (all Bonferroni-corrected p = 1.0;|rank-biserial| ≤ 0.06). Patients in the three payer groups were therefore billed comparable amounts for their trauma care. Whatever differences existed in case-mix did not translate into different bills, allowing subsequent differences in OOPE to be attributed to the payment mechanism rather than to the cost of the care delivered.

### Out-of-pocket expenditure differed markedly by payer

OOPE diverged sharply across groups despite the near-identical charges (Kruskal–Wallis H = 497.7, p < 0.001; all pairwise Bonferroni p < 0.001). Every AB-PMJAY beneficiary incurred zero OOPE (median and mean ₹0). Privately insured patients paid a median OOPE of ₹11,598 (IQR ₹5,760–₹27,508; 95% CI ₹10,529–₹13,346), or US$136. Expressed as a share of the billed amount, OOPE among these patients averaged 25.6% of the billed amount, with a median of 16.7%. In aggregate, payers met 70.0% of total charges and left the remaining 30.0% with patients. Uninsured patients paid the entire charge (median ₹85,091, or US$995; 95% CI ₹77,649–₹90,289), equal to 100% of treatment cost. Table 3 summarises charges and OOPE by payer group, and Figure 2 contrasts the similar charge distributions with the divergent OOPE distributions across groups.

**Table 3.** Total charges and out-of-pocket expenditure by payer status. Charges and OOPE in ₹ are medians with interquartile ranges. OOPE as a percentage of charge is given as the group mean with the group median in parentheses. US dollar equivalents (₹85.5 = US$1) are given in the text.

Payer group	n	Median total charge, ₹ (IQR)	Median OOPE, ₹	OOPE, % of charge: mean (median)	Zero OOPE, n (%)
AB-PMJAY	63	90,979 (57,436–118,910)	0	0 (0)	63 (100.0)
Private insurance	262	86,170 (40,351–144,149)	11,598	25.6 (16.7)	2 (0.8)
Uninsured	1,274	85,091 (37,666–145,825)	85,091	100 (100)	0 (0.0)

**Figure 2.**
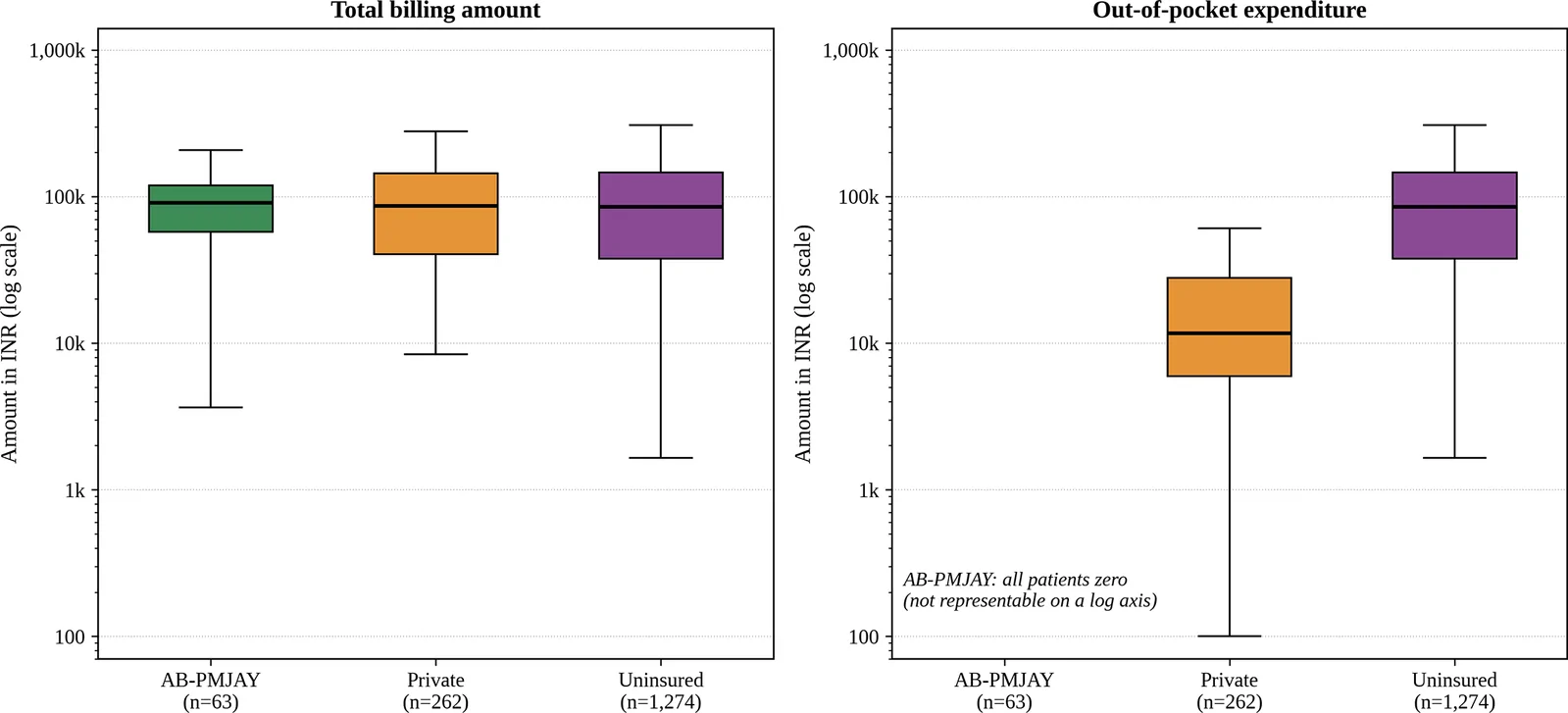
Total billing amount (left) and out-of-pocket expenditure (right) by payer group, on a logarithmic scale. Charge distributions overlap across groups; OOPE distributions diverge, from zero under AB-PMJAY to the full charge among the uninsured. Zero cannot be represented on a logarithmic axis; the AB-PMJAY group therefore has no box in the right panel, as noted on the figure.

### Catastrophic health expenditure

The share of patients facing catastrophic expenditure fell as the income threshold rose but remained heavily concentrated among the uninsured ( Figure 3). At a 10%-of-income threshold, 78.7% of uninsured patients and 22.5% of privately insured patients faced CHE, versus none under AB-PMJAY; at 25%, the figures were 50.9%, 9.9% and 0%; and at 40%, 27.9%, 5.7% and 0%. No AB-PMJAY beneficiary crossed any threshold. These estimates use per-capita income as a capacity-to-pay proxy and are indicative rather than survey-equivalent, but the gradient across payer groups is consistent at every threshold. For context, national survey analyses show catastrophic health spending in India has remained persistently high over two decades, ^4^ and the burden observed among uninsured trauma patients here is severe even against that backdrop.

**Figure 3.**
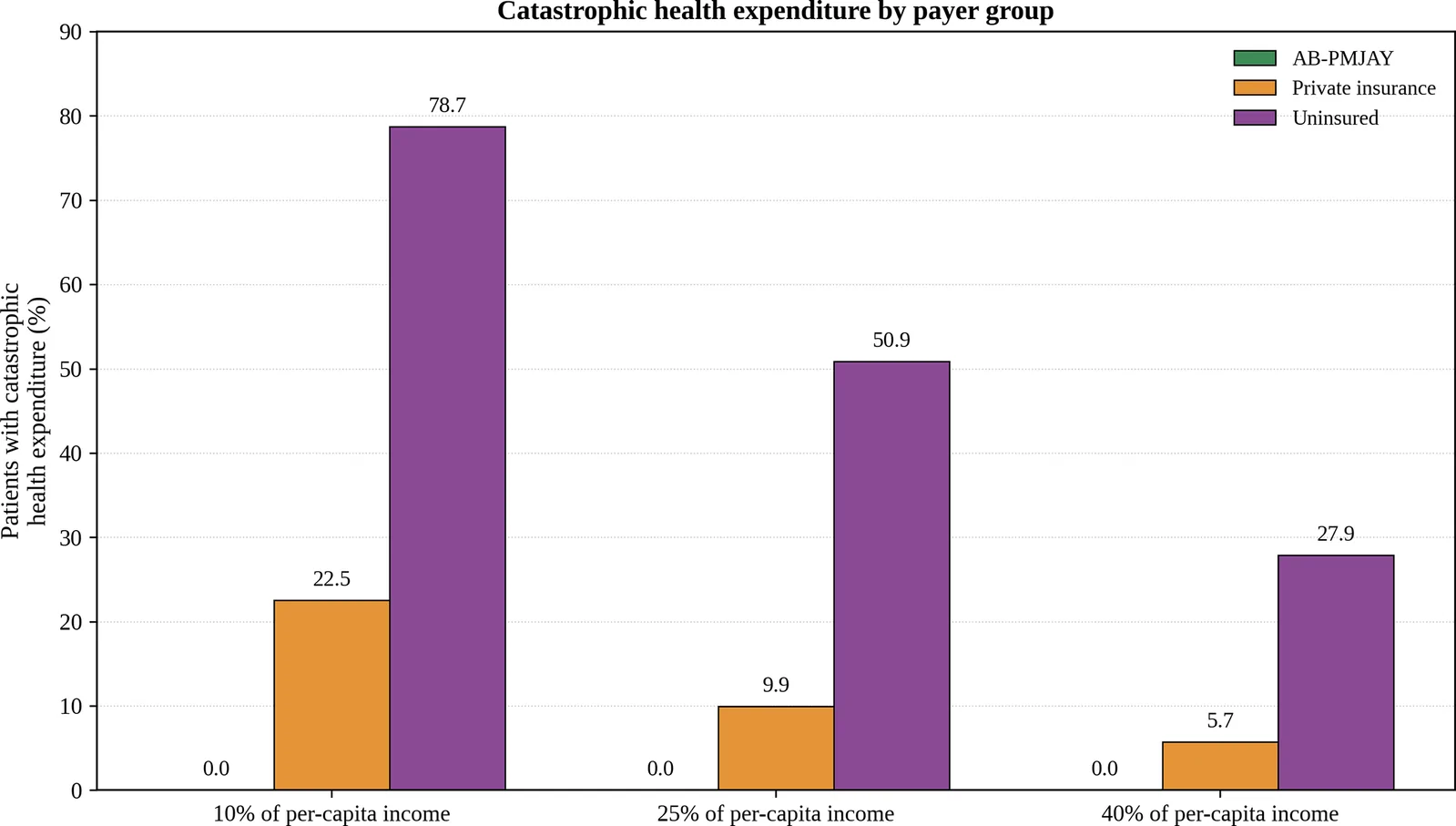
Proportion of patients facing catastrophic health expenditure at 10%, 25% and 40% of per-capita income, by payer group.

### Zero out-of-pocket expenditure

All 63 AB-PMJAY beneficiaries (100%) incurred zero OOPE. Among privately insured patients, only 2 of 262 (0.8%) achieved fully cashless treatment, and none of the uninsured did. Complete financial protection was therefore universal under AB-PMJAY and rare under private insurance.

### Variation across private insurers

Among privately insured patients, residual OOPE varied widely by insurer ( Figure 4). The 262 patients were spread across 22 insurers, third-party administrators and schemes (supplementary Table S5). Among insurers with at least three cases, mean OOPE ranged from 14.7% to 36.4% of charges; the twelve insurers with fewer than three patients, pooled, averaged 12.0%. Individual cases ranged from full reimbursement (0% OOPE) to no realised benefit (100%). The largest payers—Medicare (n = 86), Sampoorna Suraksha (n = 65), Star Health (n = 37) and Medi Assist (n = 29)—showed mean OOPE of 22.6%, 36.4%, 25.8% and 18.0% respectively. Sampoorna Suraksha is not a commercial indemnity product but a community-based mutual-aid scheme, and it recorded the highest mean OOPE of any payer with at least three cases. This dispersion indicates inconsistent benefit design, sub-limits, co-payments and exclusions across private products rather than a uniform level of protection.

**Figure 4.**
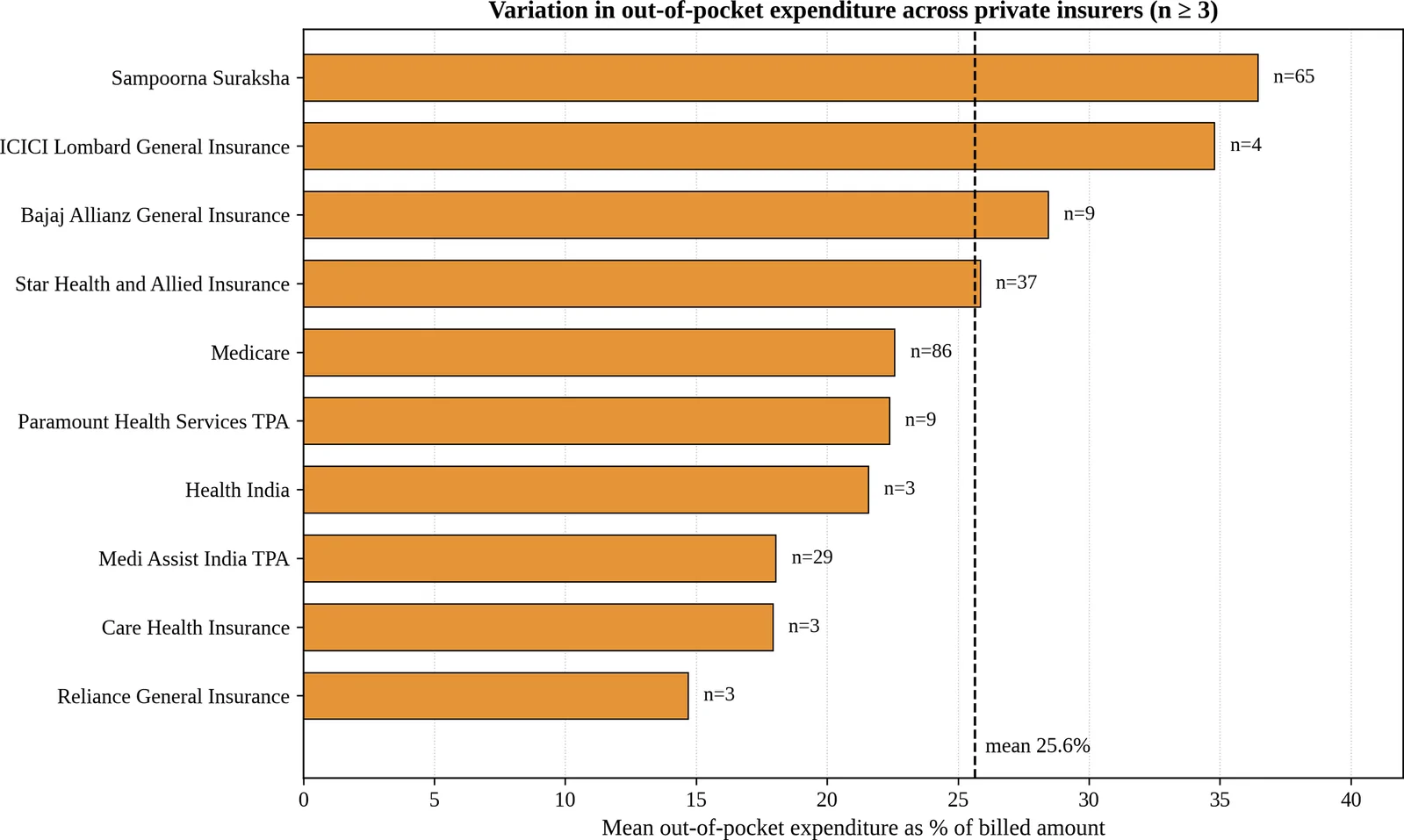
Mean out-of-pocket expenditure as a percentage of billed amount across private insurers with at least three patients; the dashed line marks the group mean (25.6%). The twelve insurers with fewer than three patients are not shown separately and are pooled in supplementary Table S5.

## Discussion

For identical care at identical prices, the financial fate of a trauma patient in this cohort was decided by a single factor: the arrangement under which they paid. Publicly funded insurance delivered complete financial risk protection: zero out-of-pocket expenditure for every beneficiary. Private insurance delivered partial and, more importantly, unpredictable protection. No insurance delivered none. This ordering—public pooling protecting fully, private insurance partially, self-payment not at all—is the same ordering we observed among 2,715 COVID-19 inpatients at this institution, where OOPE ran 0%, 21.2% and 100% across the three groups. ^13^ That the ordering reproduces across two unrelated disease contexts, a pandemic and routine road trauma, suggests it is a property of the financing mechanisms themselves rather than of any particular clinical episode; the same three-group ordering was seen for obstetric and gynaecological surgery at this centre. ^16^ Because the total charges were statistically indistinguishable across the three groups, the usual alternative explanation—that the differences in OOPE simply track differences in the size of the bill—is ruled out by the data themselves. The groups were not clinically identical: uninsured patients were somewhat older, and neurosurgical cases were far commoner among them than under AB-PMJAY ( Table 2). But the quantity that determines OOPE is the charge, and on charges the groups converge; whatever case-mix differences exist, they did not translate into different bills, and discharge outcomes were uniformly favourable across the three groups ( Table 2). This is as close to a like-for-like test of health financing mechanisms as routinely collected hospital records permit. The corollary matters for policy: complete protection is not aspirational here, it was delivered, in full, to every beneficiary who reached the scheme. What failed the uninsured was not the design of the cover but the fact that they did not have it.

The most consequential number in this study is not the OOPE gradient but its denominator: four out of five trauma patients (79.7%) arrived with no financial protection of any kind, and half of them went on to incur expenditure exceeding a quarter of annual per-capita income. Two explanations are plausible, and they are not mutually exclusive. Some of these patients belong to the ‘missing middle’: insufficiently poor for AB-PMJAY, insufficiently affluent for private cover. ^7^ Others are likely to be AB-PMJAY-eligible but unidentified: in a six-state survey of eligible households, only 62% were aware of the scheme at all, and only about three-quarters of those knew of their own eligibility ^17^—and identification is hardest at precisely the moment of unplanned emergency admission, when patients may carry no documentation and verification competes with acute care. This yields a testable proposition: if even a modest fraction of the 1,274 uninsured patients here were eligible but unidentified, on-site eligibility verification in the emergency department would have eliminated their OOPE entirely, as it did for every identified beneficiary. Quantifying the eligible-but-unenrolled fraction among self-paying trauma patients is the immediate research priority this study points to.

The pattern is not new; it holds across diseases and settings. The most directly comparable injury study, by Prinja and colleagues in North Indian public hospitals, reported CHE in 22.2% of injury-affected households and impoverishment in 12.2% at a 30%-of-consumption threshold. ^12^ Our uninsured estimates are higher (78.7% at the 10%-of-income threshold; 27.9% at 40%), but the comparison is inexact: Prinja measured household consumption in lower-tariff public hospitals, whereas we measure single-episode OOPE against per-capita income in a private tertiary centre. The shared conclusion is that uninsured injury patients face a high probability of financial catastrophe. Hospital-based studies elsewhere point the same way where pooled financing for road-trauma care is absent, though at different magnitudes: 67% of households faced catastrophic expenditure in Addis Ababa, and 19% in Ouagadougou, where under 5% of trauma patients held any insurance. ^18^ ^,^ ^19^ Earlier quasi-experimental evaluations of publicly funded insurance in India found similar, though less complete, reductions in OOPE and improvements in access. ^20^ ^,^ ^21^

Among the privately insured, the mean residual payment of 25.6% understates the real failure, which is unpredictability. The median patient paid 16.7% of charges, but the 90th percentile paid 65.5%, nearly one in five paid more than 40%, and 3% received no realised benefit at all, while only 0.8% obtained fully cashless care (supplementary Table S6). Insurance whose realised benefit ranges from complete to nil, for the same emergency at the same hospital, fails ex ante as insurance: households cannot know their exposure before the event. Part of that dispersion is structural rather than idiosyncratic: the highest-OOPE payer here was a community-based mutual-aid scheme covering self-help-group households, so voluntary cover performed worst for the members least able to absorb the residual. This dispersion, consistent with earlier Indian evidence on incomplete private protection, ^10^ points to benefit design: co-payments, room-rent-linked proportional deductions, sub-limits and consumables exclusions that trauma care triggers unpredictably. Standardising benefit packages, capping deductions for emergency admissions and prospective payment approaches such as diagnosis-related groups would make protection predictable, which is the property this market currently lacks. ^22^

India has since acted on the gap these data identify. In May 2025 the Ministry of Road Transport and Highways notified the Cashless Treatment of Road Accident Victims Scheme under section 162 of the Motor Vehicles Act, 1988; it was launched in February 2026 as the Prime Minister–Road Accident Victims’ Hospitalisation and Assured Treatment (PM-RAHAT) scheme. Any person injured in a motor vehicle crash on any road is entitled to cashless treatment of up to ₹1.5 lakh (₹150,000; about US$1,754) for seven days at a designated hospital, financed from a Motor Vehicle Accident Fund, with no insurance requirement of any kind. ^23^ Entitlement replaces identification: the 1,274 uninsured patients in this cohort would not have needed to be located in a beneficiary database, only recognised as crash victims. Our data indicate how far that entitlement reaches, and where it stops. The median uninsured charge of ₹85,091 is 57% of the ceiling, so the median uninsured patient would have been covered in full. But 302 of the 1,274 uninsured patients (23.7%) were billed above ₹1.5 lakh, and those who crossed the ceiling crossed it heavily: their mean residual liability was ₹129,254, close to the value of the cover itself. Across all uninsured admissions the ceiling would have met 73.7% of charges and left 26.3% with patients. Catastrophic expenditure would have fallen sharply without disappearing: at a 10%-of-income threshold the affected share falls from 78.7% to 16.8%, at 25% from 50.9% to 11.0%, and at 40% from 27.9% to 7.8% (supplementary Table S7). The seven-day limit is a second constraint these records cannot quantify, because length of stay was not captured. PM-RAHAT therefore converts the financing problem in emergency trauma from one of identification into one of adequacy: the entitlement is universal, the ceiling is not. Whether ₹1.5 lakh and seven days are calibrated to the observed cost distribution of trauma admissions is now the empirical question, and hospital billing data of the kind used here are the natural instrument for answering it.

### Strengths and limitations

Strengths include the use of complete, real-world billing records for all eligible admissions over a full year at a high-volume centre, a large sample, and a design that holds the institution and tariff constant across payer groups, allowing charges to be compared directly. The insurer-level analysis adds granularity rarely reported. Limitations are important. First, this is a single-centre study, so the payer mix and absolute amounts may not generalise to public hospitals or other settings, although the direction of the payer effect is likely robust. Second, we lacked household consumption data, so catastrophic expenditure is estimated against a per-capita income proxy and is indicative rather than equivalent to survey-based CHE or impoverishment measures. Third, routinely collected records did not capture injury severity, length of stay or intensive-care use, and the payer groups differed in age and in clinical department ( Table 2), with neurosurgical cases concentrated among the uninsured. Total charges were nonetheless statistically indistinguishable across groups, so these differences did not translate into different bills; but because charges rather than clinical acuity were the comparison variable, residual differences in severity cannot be excluded. Fourth, indirect costs—lost income, caregiving and rehabilitation—were not captured, so the total economic burden is underestimated. Fifth, road-user category was recorded as other or unspecified in 36.2% of admissions (579 of 1,599), largely because the external-cause code frequently records only that a collision occurred between two vehicle types without stating which the patient occupied, and the study period precedes the national cashless trauma entitlement, so the payer distribution reported here describes the position immediately before that entitlement took effect rather than the position today. Sixth, the privately insured group is heterogeneous, combining commercial indemnity products with a community-based mutual-aid scheme, so its group mean averages over materially different benefit designs. These constraints motivate multi-site, prospectively linked studies incorporating household economic data.

## Conclusions

Among road traffic accident patients treated under identical institutional conditions, out-of-pocket expenditure was determined by the payment mechanism rather than by the cost of care. AB-PMJAY provided complete financial protection, private insurance provided partial and inconsistent protection, and uninsured patients—the large majority—bore the full cost. These data describe the position immediately before a universal cashless entitlement was extended to every road crash victim in India. ^23^ They also mark its limit: nearly a quarter of the uninsured patients here (23.7%) were billed above the scheme’s ₹1.5 lakh ceiling, by a mean of ₹129,254. Calibrating that ceiling to the observed cost distribution of trauma admissions, and standardising private insurance benefit design, are the practical steps toward equitable financial protection for trauma patients in India.

## Ethics and consent

Ethical approval for this study was obtained from the Institutional Ethics Committee – 2 (Student Research), Kasturba Medical College and Kasturba Hospital, Manipal (reference IEC2: 703/2024; DHR registration EC/NEW/INST/2024/KA/0509), approved on 30 December 2024. Permission to access patient records held by the Medical Records Department was granted by the Medical Superintendent of Kasturba Hospital, who is the custodian of those records; that permission was submitted to the Institutional Ethics Committee as part of the approval process. Individual informed consent was not required, as the study was a retrospective analysis of de-identified, routinely collected billing and admission records involving no patient contact and no intervention. The study was conducted in accordance with the Declaration of Helsinki.

## Data Availability

Figshare: Same hospital, same price, different protection: out-of-pocket expenditure for emergency trauma under public insurance, private insurance and self-payment in India. https://doi.org/10.6084/m9.figshare.33183602.v2 ^24^ This project contains the following underlying data: RTA_OOPE_patient_level.xlsx. Patient-level billing and admission records for all 1,599 road traffic accident admissions analysed. The file contains, for each admission, a sequential study identifier, payer group, insurer or third-party administrator name where applicable, age in years, clinical department and hospital department code, ICD-10 external cause description, discharge outcome, total billed amount, amount met by the payer, out-of-pocket expenditure, and out-of-pocket expenditure as a percentage of the billed amount. A codebook giving variable definitions and permitted values is included in the same file. The data are anonymised: hospital account numbers, patient names, all calendar dates, addresses and contact details have been removed, and no personal identifying information is included. Figshare: Same hospital, same price, different protection: out-of-pocket expenditure for emergency trauma under public insurance, private insurance and self-payment in India. https://doi.org/10.6084/m9.figshare.33183602.v2 ^24^ This project contains the following extended data: Supplementary_tables_S1_S7.xlsx. Table S1, study flow and exclusions. Table S2, baseline characteristics by payer status. Table S3, total charges and out-of-pocket expenditure by payer. Table S4, catastrophic health expenditure at 10%, 25% and 40% thresholds. Table S5, out-of-pocket expenditure by private insurer and third-party administrator. Table S6, distribution of realised benefit among privately insured patients. Table S7, counterfactual application of the 2025 cashless trauma entitlement ceiling to the uninsured group. STROBE_checklist.docx. Completed STROBE checklist for cross-sectional studies. Data are available under the terms of the Creative Commons Attribution 4.0 International license (CC-BY 4.0).
