# Same Hospital, Same Tariff, Different Protection: Out-of-pocket Expenditure among Road Traffic Accident Admissions under Public Insurance, Private Insurance and Self-payment in India

**DOI:** 10.12688/f1000research.188694.2

**Published:** 2026-11-06

**Authors:** Rajesh Kamath, Amisha Thakar, Ravichandran Nair, Akhila Doddamani, Reena Verma, R. Somasundaram, Anindita Ghosh

**Affiliations:** 1 Department of Healthcare and Hospital Management, Prasanna School of Public Health, Manipal Academy of Higher Education, Manipal, India; 2 Department of Social and Health Innovation, Prasanna School of Public Health, Manipal Academy of Higher Education, Manipal, India; 3 Department of Community Medicine, Kasturba Medical College, Manipal Academy of Higher Education, Manipal, India; 4 School of Nutrition and Dietetics, Symbiosis Skills and Professional University, Pimpri-Chinchwad, Maharashtra, 412101, India; 5 College of Management, SRM Institute of Science and Technology (Deemed to be University) - Tiruchirappalli Campus, Tiruchirappalli, Tamil Nadu, 621105, India

**Keywords:** out-of-pocket expenditure; road traffic accidents; AB-PMJAY; private health insurance; financial risk protection; catastrophic health expenditure; India

## Abstract

**Background:**

Out-of-pocket expenditure (OOPE) finances 43.4% of total health expenditure in India, and emergency trauma—unforeseeable, unpostponable and costly—is among the sharpest tests of whether a health-financing mechanism protects households from catastrophic spending. Insured and uninsured patients are normally treated in different facilities at different tariffs, confounding the effect of the payment mechanism with the setting in which care is given.

**Methods:**

In this retrospective cross-sectional study, we compared OOPE across three financing arrangements—Ayushman Bharat–Pradhan Mantri Jan Arogya Yojana (AB-PMJAY), private health insurance and self-payment—using billing records for 1,599 of 1,711 consecutive road traffic accident admissions to a tertiary trauma centre in coastal Karnataka, India (July 2023–July 2024). All groups were billed under the same tariff. Charges were compared with and without adjustment for age, department and road-user category. OOPE was the patient payment recorded in hospital billing.

**Results:**

Of 1,599 patients, 63 (3.9%) were AB-PMJAY beneficiaries, 262 (16.4%) were privately insured and 1,274 (79.7%) were uninsured. Median total charges were similar (₹90,979, ₹86,170 and ₹85,091; Kruskal–Wallis p = 0.70), including after adjustment (ratio of medians against the uninsured 0.95, 95% CI 0.85–1.08, for AB-PMJAY; 1.02, 0.89–1.14, for private insurance). No AB-PMJAY beneficiary had any recorded hospital OOPE. Privately insured patients paid a median of ₹11,598 (mean 25.6% of charges), highest under the community mutual-aid scheme (mean 36.4% of charges). Uninsured patients paid the entire charge. OOPE exceeded 10% of per-capita income for 0%, 22.5% and 78.7% of the three groups.

**Conclusions:**

Recorded OOPE followed payer status while charges did not. Injury severity and household means were unobserved, so this is an association, not a causal effect. Four-fifths of admissions had no cover registered with the hospital. India’s 2025 cashless trauma entitlement addresses that gap, but 23.7% of uninsured admissions here were billed above its ₹1.5 lakh ceiling.

## Introduction

Out-of-pocket expenditure (OOPE) finances 43.4% of total health expenditure in India. The share has fallen from 64.2% a decade earlier, but it remains among the highest in the world, and in absolute terms household spending continues to rise. ^1^ The decline has not been monotonic: the share rose from 39.4% in 2021–22 to 43.4% in 2022–23, a four-percentage-point reversal in a single year. ^1^ Direct payment at the point of care is the dominant mode of health financing, and its consequence is well established: when a household meets a large medical bill from its own pocket, that spending can become catastrophic—conventionally, OOPE exceeding a fixed share of the household’s capacity to pay—and can push families below the poverty line. ^2^ ^,^ ^3^ Financial risk protection, the pooling of health risk so that illness does not translate into impoverishment, is the central objective of Universal Health Coverage and of Sustainable Development Goal target 3.8 (indicator 3.8.2). India’s progress on it remains halting. ^4^

The instrument through which a health system delivers financial protection is the payment mechanism: how a given admission is financed determines how much of its cost the household ultimately bears. India is expanding two pooling mechanisms at once. Ayushman Bharat–Pradhan Mantri Jan Arogya Yojana (AB-PMJAY), the world’s largest publicly funded health assurance scheme, offers cashless cover of up to ₹5 lakh (₹500,000; about US$5,850) per family per year to some 550 million individuals in the poorest two-fifths of the population, and since October 2024 to all citizens aged 70 years and above irrespective of income ^5^; commercial private insurance grows among wealthier households, alongside community-based and mutual-aid schemes that extend voluntary cover to poorer ones. ^6^ Between them, at least 30% of Indians—the ‘missing middle’—hold neither. ^7^ Whether these mechanisms actually protect households, and how completely, is a first-order empirical question for Indian health financing.

Emergency trauma is the sharpest test of that protection. The need for care is unforeseeable, unpostponable and expensive; India recorded 177,175 road-crash deaths and 471,441 recorded injuries in 2024, disproportionately among young, economically productive adults. Crash severity is rising: deaths per 100 crashes climbed from 21.6 in 2005 to 36.3 in 2024, so the financing question is increasingly about the cost of surviving a crash rather than the cost of preventing one. ^8^ ^,^ ^9^ Yet the evidence on how payment mechanisms perform is weak, because comparisons across payer groups are almost always confounded by setting: insured and uninsured patients are drawn from different facilities with different tariffs, case-mix and quality, ^10^ so the protective effect of the mechanism cannot be separated from where and how patients were treated. Injury-related hospitalisation in India consistently shows heavy OOPE, with catastrophic spending affecting roughly one in five injury-affected households and lack of insurance its most consistent determinant. ^11^ ^,^ ^12^ For unplanned emergency admissions the confounding is especially severe: trauma patients, unlike elective patients, cannot choose their hospital.

This study narrows that confounding by design. We analysed billing records for all eligible road traffic accident admissions with complete billing over one year at a single tertiary trauma centre where AB-PMJAY beneficiaries, privately insured and uninsured patients are treated by the same clinical teams at the same tariff. Holding the institution and tariff constant removes differences in price between facilities. It does not remove differences in case-mix within the facility, which we partly measure and adjust for. This design extends our earlier finding among 2,715 COVID-19 inpatients at the same institution—where OOPE ran 0% under AB-PMJAY, 21.2% under private insurance and 100% for the uninsured ^13^—from a pandemic-specific shock to routine emergency trauma, testing whether the ordering of financing mechanisms holds across disease contexts. We estimate recorded hospital OOPE among road traffic accident admissions, compare it across the three mechanisms, and examine the heterogeneity of protection within private insurance.

## Methods

### Study design and setting

We conducted a retrospective, single-centre, cross-sectional analysis of hospital billing and admission records. The unit of analysis is the admission, observed once from admission to discharge. The study site is a National Accreditation Board for Hospitals and Healthcare Providers (NABH)-accredited tertiary care teaching hospital of approximately 2,000 beds in coastal Karnataka, India, functioning as a major regional trauma referral centre and treating a high volume of emergency cases. Reporting follows the STROBE guideline for cross-sectional studies ^14^; the completed checklist is provided as extended data and each item is addressed in the relevant subsection below.

### Participants and eligibility

The study was scoped to three financing arrangements for emergency trauma care: AB-PMJAY, private health insurance, and self-payment. Patients admitted through the emergency medicine department following a road traffic accident between July 2023 and July 2024 were eligible if their inpatient care was financed through one of these three modes. Of 1,711 road traffic accident admissions in the period, 1,609 met this criterion. After exclusion of 10 privately insured admissions whose billing records were too incomplete to permit accurate OOPE calculation, 1,599 were classified into three mutually exclusive groups according to the payer recorded against the admission in the hospital billing system: AB-PMJAY beneficiaries, privately insured patients, and uninsured (self-paying) patients. Private health insurance was defined as any non-government arrangement, registered with the hospital for the admission, under which a third-party payer was liable to settle the bill directly with the hospital. Admissions stayed in this group whatever the outcome of the claim. Where nothing was received from the registered payer, whether because a claim was rejected or because the patient settled the bill and claimed from the payer directly, the recorded patient payment equalled the full charge; this applied to 8 of the 262 privately insured admissions. Any reimbursement a patient received after discharge is not visible in hospital records. Patients who registered no cover with the hospital were recorded as self-paying. The private category is heterogeneous. It comprises commercial insurers and their third-party administrators (n = 111, 20 payers); Medicare, a health scheme operated by the university with which the hospital is affiliated (n = 86); and the Sampoorna Suraksha programme of the Shri Kshethra Dharmasthala Rural Development Project, a community-based mutual-aid scheme financed by subscriptions from self-help-group households (n = 65). The three were analysed as one group because each is a non-government arrangement under which a third party settles the bill directly with the hospital, the mode of financing the study contrasts with AB-PMJAY and self-payment; results are also reported by type of payer. The remaining 102 patients fell outside the study’s scope: they were financed through arrangements outside the three study modes—Employees’ State Insurance (n = 75), the Ex-servicemen Contributory Health Scheme (n = 6), public-sector-undertaking employer schemes (n = 3), a state government scheme (n = 13), a public-sector third-party administrator (n = 3) and free-ward care (n = 2)—whose itemised billing was not extracted and whose financing rules differ from the three modes of interest. The 102 include one record initially segregated into the private insurance group during data preparation that was, on verification of the payer name against the hospital billing system, an employer scheme of a public-sector undertaking: it was reclassified to the other-schemes group before analysis. The analysed set comprised inpatient admissions with complete finance-department billing records. Patients declared dead on arrival, and those who died during emergency-department care before inpatient admission, are not represented in this extract; their medico-legal and administrative billing is handled separately from routine inpatient care. Patients treated in the emergency department and discharged without admission were outside the eligibility criterion.

### Variables and outcome measures

The primary outcome was recorded hospital OOPE: the amount the hospital billing system recorded as paid by the patient for the admission, after any payment by the registered payer, expressed both in Indian Rupees (₹) and as a percentage of the total charge. Total charge was the gross amount billed for the admission at the hospital’s standard tariff, before any payer-specific settlement, deduction or discount; the same tariff applied to all three groups. For uninsured patients OOPE equalled the total charge. For privately insured admissions the records give the amount settled by the payer, but do not itemise deductions, co-payments, exclusions or disallowed amounts, so these components cannot be separated. For AB-PMJAY admissions the finance department recorded the gross bill and the patient’s payment, and the amount met by the scheme was taken as their difference. OOPE was therefore observed in every group, not assumed. The amount actually reimbursed to the hospital by the State Health Agency at the scheme’s package rates was not part of the extract; any difference between that reimbursement and the gross bill was not recorded against the patient. Payments made outside the hospital billing system—medicines, diagnostics or consumables bought elsewhere, transport and follow-up care—were not captured, and the outcome is accordingly described throughout as recorded hospital OOPE. Secondary outcomes were the total charge, the proportion of patients with zero recorded OOPE, OOPE relative to per-capita income, and payer-level variation in OOPE among privately insured patients. Amounts were converted to US dollars at ₹85.5 = US$1, the rate prevailing at the time of analysis.

### Data sources

Data were extracted from the hospital’s finance department (itemised billing, amounts settled by payers and patient payments) and medical records/information-technology systems (admission details and payer identity), and linked at patient level by inpatient number. All records were de-identified before analysis; direct identifiers were removed and access was restricted to the investigators.

### OOPE relative to per-capita income

Catastrophic health expenditure (CHE) is conventionally defined at the household level, as OOPE exceeding a fixed share of household consumption expenditure or capacity to pay. ^2^ ^,^ ^3^ Household consumption data were not available for these patients, so household CHE could not be measured. In its place we report an admission-level indicator of potential financial hardship: the proportion of patients whose recorded OOPE for the admission exceeded 10%, 25% and 40% of Karnataka’s per-capita income for 2023–24 (₹332,926, about US$3,894; Economic Survey of Karnataka). ^15^ The corresponding cut-offs are ₹33,293, ₹83,232 and ₹133,170. The thresholds follow the literature: 10% and 25% were the thresholds of Sustainable Development Goal indicator 3.8.2, applied to total household expenditure or income, ^16^ and 40% is the conventional capacity-to-pay threshold, ^2^ which the Inter-Agency and Expert Group on SDG Indicators proposed for the indicator in its 2025 comprehensive review, applied to the household’s discretionary budget. ^17^ The indicator applies one fixed denominator to every patient. It does not distinguish richer households from poorer ones, it compares a single admission with a year’s income per person rather than per household, and it cannot establish whether any household experienced financial catastrophe or impoverishment. It measures the size of a hospital bill against average income: it is not household CHE, and it is not comparable with survey-based CHE estimates. ^2^ ^,^ ^3^

### Statistical analysis

Continuous variables were summarised as medians with interquartile ranges (IQR); billing and OOPE distributions were markedly right-skewed and non-normal on the Shapiro–Wilk test (all p < 0.001). Total charges and OOPE were compared across the three groups with the Kruskal–Wallis test, followed by pairwise Mann–Whitney U tests with Bonferroni correction; rank-biserial correlation is reported as an effect size. A non-significant test does not establish that charges were equal, ^18^ and no equivalence margin was specified before analysis. We therefore report the size and precision of differences in charges: ratios and differences of medians against the uninsured group, with 95% CIs from 5,000 bootstrap resamples of admissions. Charges were then compared after adjustment for the observed differences in case-mix, using the same covariates in two models: age (linear and quadratic terms), clinical department (four categories) and road-user category (five categories). The first was a median regression of log charges, whose exponentiated payer coefficients are adjusted ratios of medians, with 95% CIs from 1,000 bootstrap resamples of admissions. The second was a generalised linear model with gamma distribution, log link and heteroskedasticity-robust standard errors, whose exponentiated coefficients are adjusted ratios of means. ^19^ Discharge outcome was not adjusted for, as it follows treatment. Two sensitivity analyses repeated the adjusted model with seven hospital-unit categories in place of four clinical departments, and after excluding neurosurgical admissions; charges were also compared within each clinical department. Among patients outside AB-PMJAY, the association of private insurance with OOPE above each income threshold was estimated as an adjusted risk ratio by Poisson regression with robust standard errors, ^20^ with the same covariates; no AB-PMJAY beneficiary crossed any threshold, so that group could not be modelled. Within private insurance, OOPE as a percentage of charge was compared across the three types of payer by the Kruskal–Wallis test and by linear regression with robust standard errors, adjusted for log charge, age and clinical department. Ninety-five per cent CIs for medians were obtained by bias-corrected and accelerated bootstrap (5,000 resamples of admissions) and for proportions by the Wilson method. Categorical variables were compared with Pearson’s χ ^2^ test where no more than 20% of cells had expected counts below five and all expected counts were two or greater; the Fisher–Freeman–Halton exact test was used otherwise, with the p-value for road-user category estimated from 200,000 Monte Carlo tables. All analyses were complete-case and were performed in Python 3 (pandas, SciPy, statsmodels) with a fixed random seed; the analysis script is deposited with the extended data (see Data availability). Two-sided p < 0.05 was considered significant. Road-user category was assigned from the ICD-10 external cause-of-injury code recorded at admission, using the code blocks directly: V01–V09 pedestrian, V10–V19 cyclist, V20–V39 motorcyclist or three-wheeler occupant, V40–V59 car, van or pick-up occupant. Codes for bus and heavy-vehicle occupants, and codes in which the injured person’s role is not specified, were grouped as other or unspecified; no case-note review was undertaken to resolve the latter.

All analyses were performed in Python 3 using the open-source pandas, SciPy and statsmodels libraries. The analysis script, RTA_OOPE_analysis.py, reproduces every estimate in the article from the underlying data and is deposited with the extended data. No proprietary software was used.

## Results

### Study population and payer mix

A total of 1,599 RTA patients met the eligibility criteria ( Figure 1). Of these, 63 (3.9%) were covered by AB-PMJAY, 262 (16.4%) by private health insurance, and 1,274 (79.7%) were uninsured ( Table 1). Uninsured patients thus accounted for almost four-fifths of trauma admissions, underscoring the continued predominance of direct payment in emergency trauma financing at this centre.

**Figure 1.**
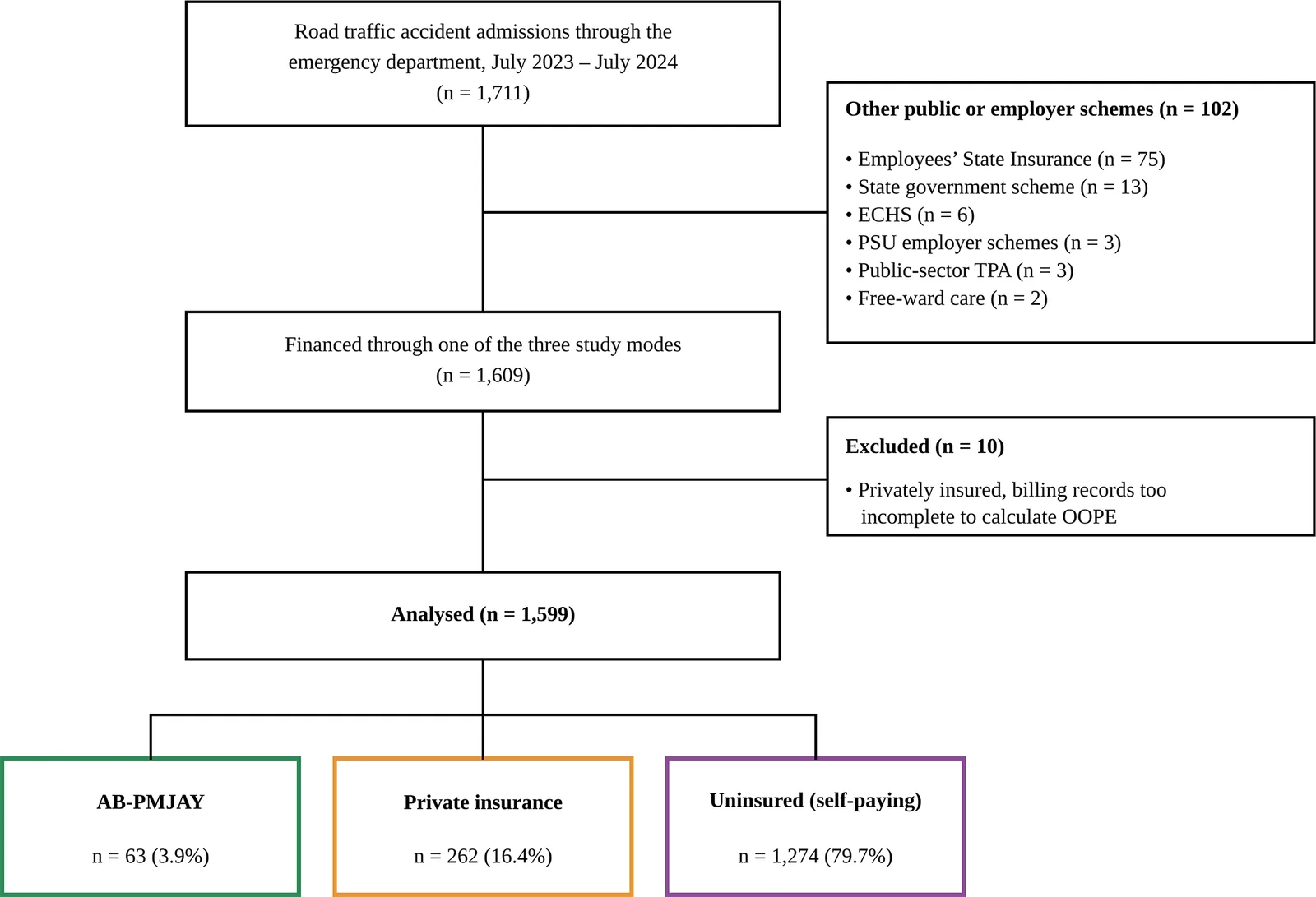
Study flow diagram. ECHS, Ex-servicemen Contributory Health Scheme; PSU, public-sector undertaking; TPA, third-party administrator.

**Table 1.** Distribution of road traffic accident patients by payer status (n = 1,599).

Payer group	Patients, n	Proportion, %
AB-PMJAY	63	3.9
Private health insurance	262	16.4
Uninsured	1,274	79.7
Total	1,599	100.0

### Age and case-mix differed across payer groups

The three groups were not identical in composition ( Table 2). Uninsured patients were older (median 39 years, IQR 28–52) than AB-PMJAY (34, 24–48) and privately insured patients (34, 23–45), a difference that was statistically significant (Kruskal–Wallis p < 0.001) though modest in magnitude. Case-mix also differed by clinical department (χ ^2^ = 37.4, p < 0.001): orthopaedics was the commonest department in every group, but neurosurgery accounted for almost a third of uninsured admissions (32.8%) and under a quarter of privately insured admissions (23.3%) but only 3.2% under AB-PMJAY. Road-user category also differed across groups (Fisher–Freeman–Halton exact p = 0.003): motorcyclists and three-wheeler occupants were the largest category throughout, accounting for 57.4% of all admissions, but ranged from 47.6% under AB-PMJAY to 62.6% among privately insured patients. Discharge outcomes were favourable in all three groups: 1,554 patients (97.2%) were recorded as improved or recovered, and no in-hospital deaths appear in the analysed set. The distribution of non-favourable outcomes—unchanged, worse, discharge against medical advice, or diagnosis only—did differ across payer groups (5, 3 and 37 patients, or 7.9%, 1.1% and 2.9%; Fisher–Freeman–Halton exact p = 0.018), but the counts are small and the gradient does not run against the uninsured. The groups therefore differed in case-mix. The next section asks whether those differences translated into different charges.

**Table 2.** Baseline characteristics by payer status: age, clinical department, road-user category and discharge outcome. Other surgical comprises oral, hand, general and plastic surgery; medical/other comprises general medicine, pulmonology, ophthalmology and other units. Road-user category is derived from ICD-10 external-cause-of-injury codes: pedestrian, V01–V09; cyclist, V10–V19; motorcyclist or three-wheeler occupant, V20–V39; car, van or pick-up occupant, V40–V59; other or unspecified comprises bus and heavy-vehicle occupants and all codes in which the injured person’s role is not specified.

Characteristic	AB-PMJAY (n = 63)	Private insurance (n = 262)	Uninsured (n = 1,274)
**Age, years, median (IQR)**	34 (24–48)	34 (23–45)	39 (28–52)
**Clinical department, n (%)**			
Orthopaedics	39 (61.9)	124 (47.3)	526 (41.3)
Neurosurgery	2 (3.2)	61 (23.3)	418 (32.8)
Other surgical	22 (34.9)	64 (24.4)	284 (22.3)
Medical/other	0 (0.0)	13 (5.0)	46 (3.6)
**Road-user category, n (%)**			
Motorcyclist or three-wheeler occupant	30 (47.6)	164 (62.6)	724 (56.8)
Car, van or pick-up occupant	1 (1.6)	10 (3.8)	53 (4.2)
Pedestrian	1 (1.6)	6 (2.3)	23 (1.8)
Cyclist	1 (1.6)	5 (1.9)	2 (0.2)
Other or unspecified	30 (47.6)	77 (29.4)	472 (37.0)
**Discharge outcome, n (%)**			
Improved or recovered	58 (92.1)	259 (98.9)	1,237 (97.1)
Other (unchanged, worse, against advice or diagnosis only)	5 (7.9)	3 (1.1)	37 (2.9)

### Total charges were similar across payer groups, before and after adjustment

Median total charges were closely similar across the three groups ( Table 3): ₹90,979 (IQR ₹57,436–₹118,910; 95% CI ₹77,148–₹109,334) for AB-PMJAY, ₹86,170 (IQR ₹40,351–₹144,149; 95% CI ₹73,950–₹98,104) for privately insured, and ₹85,091 (IQR ₹37,666–₹145,825; 95% CI ₹77,707–₹90,283) for uninsured patients. The Kruskal–Wallis test showed no difference in charges across groups (H = 0.72, p = 0.70), and all pairwise comparisons were non-significant (all Bonferroni-corrected p = 1.0; |rank-biserial| ≤ 0.06). Against the uninsured, the ratio of median charges was 1.07 (95% CI 0.92–1.29) for AB-PMJAY and 1.01 (0.85–1.18) for private insurance ( Table 4). Adjustment for age, clinical department and road-user category changed little: the adjusted ratio of medians was 0.95 (0.85–1.08) for AB-PMJAY and 1.02 (0.89–1.14) for private insurance, and the adjusted ratio of means 0.86 (0.71–1.03) and 0.99 (0.86–1.15). After adjustment, the data are compatible with median AB-PMJAY charges up to 15% lower or 8% higher than uninsured charges, and with median private charges up to 11% lower or 14% higher. The excess of neurosurgical admissions among the uninsured did not inflate their bills: among uninsured patients, the median neurosurgical charge (₹51,016) was less than half the median orthopaedic charge (₹109,016). Within orthopaedics, the largest department, median charges were ₹111,097, ₹117,323 and ₹109,016 across the three groups (p = 0.56; Table 4). Within neurosurgery, charges did differ (p = 0.034): the median was ₹39,045 for privately insured and ₹51,016 for uninsured patients, and only two AB-PMJAY beneficiaries were neurosurgical admissions. One sensitivity analysis pointed the other way. With neurosurgical admissions excluded, mean AB-PMJAY charges were 20% lower than uninsured charges after adjustment (ratio of means 0.80, 0.69–0.92), while private charges were unchanged (1.02, 0.90–1.15); medians did not differ (Kruskal–Wallis p = 0.35). Charges were therefore similar across payer groups within the precision these data allow. They were not shown to be identical, and the comparisons that follow carry that qualification.

**Table 3.** Total charges and recorded out-of-pocket expenditure by payer status. Total charge is the gross amount billed at the hospital tariff; OOPE is the patient payment recorded in hospital billing. Charges and OOPE in ₹ are medians with interquartile ranges. OOPE as a percentage of charge is given as the group mean with the group median in parentheses. Ninety-five per cent confidence intervals are given in Tables 4 and 5 and in the text. US dollar equivalents (₹85.5 = US$1) are given in the text.

Payer group	n	Median total charge, ₹ (IQR)	Median OOPE, ₹	OOPE, % of charge: mean (median)	Zero OOPE, n (%)
AB-PMJAY	63	90,979 (57,436–118,910)	0	0 (0)	63 (100.0)
Private insurance	262	86,170 (40,351–144,149)	11,598	25.6 (16.7)	2 (0.8)
Uninsured	1,274	85,091 (37,666–145,825)	85,091	100 (100)	0 (0.0)

**Table 4.** Total charges by payer status: size of differences, adjusted comparisons and comparisons within clinical department. Panel A gives ratios and differences against uninsured patients (reference), with 95% confidence intervals. Unadjusted ratios and differences of medians use 5,000 bootstrap resamples of admissions. Adjusted ratios of medians are from median regression of log charges (1,000 bootstrap resamples); ratios of means are from a gamma generalised linear model with log link and robust standard errors. Adjustment is for age (linear and quadratic), clinical department (four categories) and road-user category (five categories); the first sensitivity analysis replaces the four departments with seven hospital-unit categories. Panel B gives median total charges (n) by clinical department, with Kruskal–Wallis p-values across payer groups.

Measure	AB-PMJAY	Private insurance	Uninsured
**A. Comparisons with uninsured patients**			
Median total charge, ₹ (95% CI)	90,979 (77,148–109,334)	86,170 (73,950–98,104)	85,091 (77,707–90,283)
Difference in medians, ₹ (95% CI)	5,888 (−7,051 to 24,305)	1,079 (−13,303 to 14,708)	Reference
Ratio of medians, unadjusted	1.07 (0.92–1.29)	1.01 (0.85–1.18)	1
Ratio of medians, adjusted	0.95 (0.85–1.08)	1.02 (0.89–1.14)	1
Ratio of means, unadjusted	0.88 (0.73–1.06)	0.98 (0.86–1.12)	1
Ratio of means, adjusted	0.86 (0.71–1.03)	0.99 (0.86–1.15)	1
Ratio of means, adjusted, seven hospital-unit categories	0.91 (0.75–1.09)	1.04 (0.91–1.20)	1
Ratio of means, adjusted, excluding neurosurgery (n = 61, 201, 856)	0.80 (0.69–0.92)	1.02 (0.90–1.15)	1
**B. Median total charge, ₹ (n), by clinical department**			
Orthopaedics (p = 0.56)	111,097 (39)	117,323 (124)	109,016 (526)
Neurosurgery (p = 0.034)	334,877 (2)	39,045 (61)	51,016 (418)
Other surgical (p = 0.12)	54,179 (22)	70,852 (64)	64,228 (284)
Medical/other (p = 0.48)	— (0)	44,118 (13)	69,856 (46)

### Out-of-pocket expenditure differed markedly by payer

OOPE diverged sharply across groups despite the similar charges (Kruskal–Wallis H = 497.7, p < 0.001; all pairwise Bonferroni p < 0.001). No AB-PMJAY beneficiary had any recorded hospital OOPE (median and mean ₹0). Privately insured patients paid a median OOPE of ₹11,598 (IQR ₹5,760–₹27,508; 95% CI ₹10,529–₹13,510), or US$136. Expressed as a share of the billed amount, OOPE among these patients averaged 25.6% of the billed amount, with a median of 16.7%. In aggregate, payers met 70.0% of total charges and left the remaining 30.0% with patients. Uninsured patients paid the entire charge (median ₹85,091, or US$995), equal to 100% of the bill. Because OOPE is measured against each patient’s own bill, differences in charges of the size the confidence intervals above allow cannot produce a gradient from 0% through 25.6% to 100% of the bill. Table 3 summarises charges and OOPE by payer group, and Figure 2 contrasts the similar charge distributions with the divergent OOPE distributions across groups.

**Figure 2.**
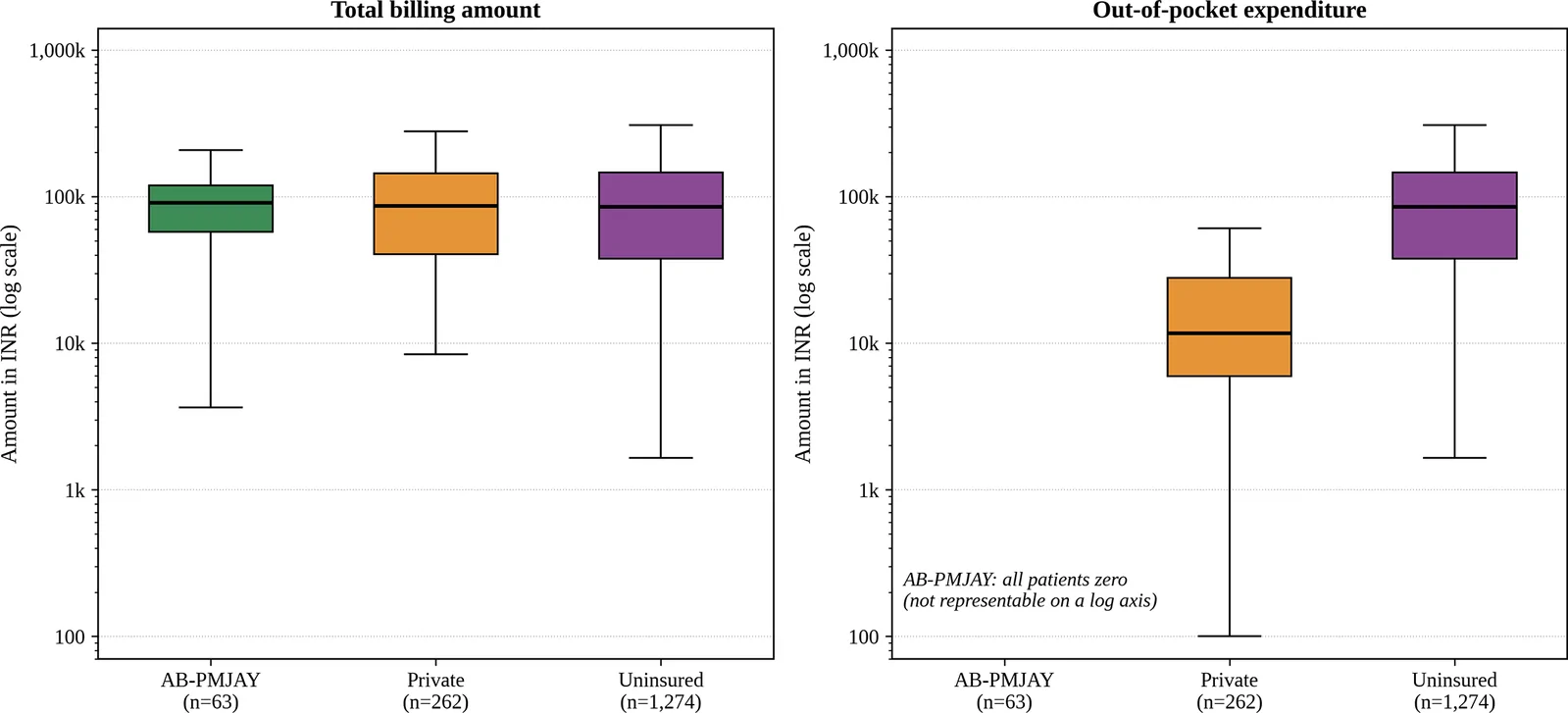
Total billing amount (left) and out-of-pocket expenditure (right) by payer group, on a logarithmic scale. Charge distributions overlap across groups; OOPE distributions diverge, from zero under AB-PMJAY to the full charge among the uninsured. Zero cannot be represented on a logarithmic axis; the AB-PMJAY group therefore has no box in the right panel, as noted on the figure. The two privately insured patients with zero OOPE are likewise not shown in the right panel. Whiskers extend to 1.5 times the interquartile range; outlying values are not shown.

### OOPE relative to per-capita income

The share of patients whose recorded OOPE exceeded each income threshold fell as the threshold rose but remained heavily concentrated among the uninsured ( Figure 3; Table 5). Above 10% of per-capita income (₹33,293), the shares were 78.7% (95% CI 76.4–80.9) of uninsured and 22.5% (17.9–28.0) of privately insured patients, as against none of the AB-PMJAY beneficiaries (0 of 63; 95% CI 0–5.7); above 25% (₹83,232), 50.9% (48.1–53.6), 9.9% (6.9–14.1) and 0%; and above 40% (₹133,170), 27.9% (25.5–30.4), 5.7% (3.5–9.2) and 0%. After adjustment for age, clinical department and road-user category, privately insured patients were less likely than the uninsured to cross each threshold: the adjusted risk ratio was 0.28 (95% CI 0.22–0.35) at 10%, 0.19 (0.13–0.27) at 25% and 0.21 (0.13–0.35) at 40%. These are admission-level indicators of potential hardship, not household CHE. National survey analyses show catastrophic health spending in India has remained persistently high over two decades ^4^; the figures here are not comparable with those estimates and are not offered as such.

**Figure 3.**
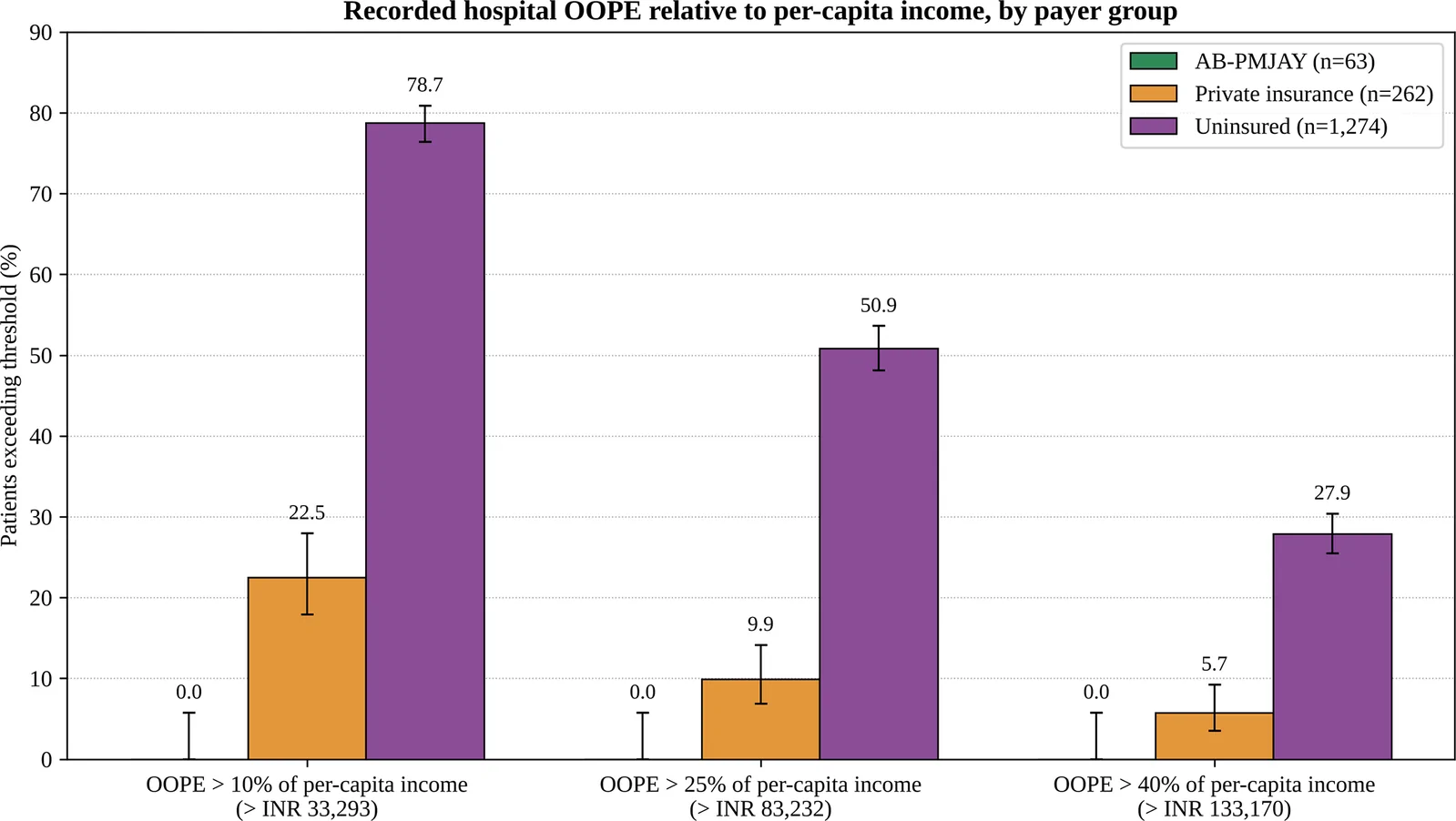
Proportion of patients whose recorded hospital out-of-pocket expenditure exceeded 10%, 25% and 40% of Karnataka’s per-capita income for 2023–24 (₹33,293, ₹83,232 and ₹133,170), by payer group. Error bars are Wilson 95% confidence intervals. This is an admission-level indicator of potential financial hardship, not household catastrophic health expenditure.

**Table 5.** Recorded hospital out-of-pocket expenditure by payer status: median, zero OOPE, and OOPE above 10%, 25% and 40% of per-capita income. Cut-offs are 10%, 25% and 40% of Karnataka’s per-capita income for 2023–24 (₹332,926): ₹33,293, ₹83,232 and ₹133,170. Cells give n/N (%; 95% CI). Confidence intervals are bias-corrected and accelerated bootstrap intervals for medians (5,000 resamples) and Wilson intervals for proportions. This is an admission-level indicator of potential financial hardship, not household catastrophic health expenditure.

Payer group	Median OOPE, ₹ (95% CI)	Zero OOPE	OOPE > 10% of income	OOPE > 25% of income	OOPE > 40% of income
AB-PMJAY (n = 63)	0	63/63 (100; 94.3–100)	0/63 (0; 0–5.7)	0/63 (0; 0–5.7)	0/63 (0; 0–5.7)
Private insurance (n = 262)	11,598 (10,529–13,510)	2/262 (0.8; 0.2–2.7)	59/262 (22.5; 17.9–28.0)	26/262 (9.9; 6.9–14.1)	15/262 (5.7; 3.5–9.2)
Uninsured (n = 1,274)	85,091 (77,707–90,283)	0/1,274 (0; 0–0.3)	1,003/1,274 (78.7; 76.4–80.9)	648/1,274 (50.9; 48.1–53.6)	355/1,274 (27.9; 25.5–30.4)

### Zero recorded hospital out-of-pocket expenditure

All 63 AB-PMJAY beneficiaries (100%; 95% CI 94.3–100) had zero recorded hospital OOPE. Among privately insured patients, only 2 of 262 (0.8%) were discharged with nothing to pay, and none of the uninsured were. Zero recorded OOPE was therefore universal under AB-PMJAY in this cohort and rare under private insurance. With 63 beneficiaries, these data cannot exclude non-zero recorded OOPE in up to about 6% of AB-PMJAY admissions at this hospital, and they say nothing about payments made outside the hospital bill.

### Variation across private payers

Among privately insured patients, residual OOPE varied widely by payer ( Figure 4). The 262 patients were spread across 22 insurers, third-party administrators and schemes (supplementary Table S5). Among payers with at least three cases, mean OOPE ranged from 14.7% to 36.4% of charges; the twelve payers with fewer than three patients, pooled, averaged 12.0%. Individual cases ranged from full payment by the payer (0% OOPE) to no payment at all (100%). The three types of private payer differed ( Table 6; Kruskal–Wallis p < 0.001). Commercial insurers and their third-party administrators (n = 111) left a mean of 21.7% of charges with patients (median 15.4%); Medicare, the university-run scheme (n = 86), 22.6% (median 13.8%); and Sampoorna Suraksha, the community mutual-aid scheme (n = 65), 36.4% (median 23.0%). After adjustment for charge, age and clinical department, members of the mutual-aid scheme paid 14.9 percentage points more of their bill than patients with commercial insurance (95% CI 6.2–23.5), while the university-run scheme did not differ from commercial insurance (3.3 points; −2.8 to 9.4). Five of the eight privately insured patients with no payer contribution were members of the mutual-aid scheme. Estimates for payers with few patients are exploratory. The dispersion is consistent with differences in benefit design, sub-limits, co-payments and exclusions across products, which these records do not itemise.

**Figure 4.**
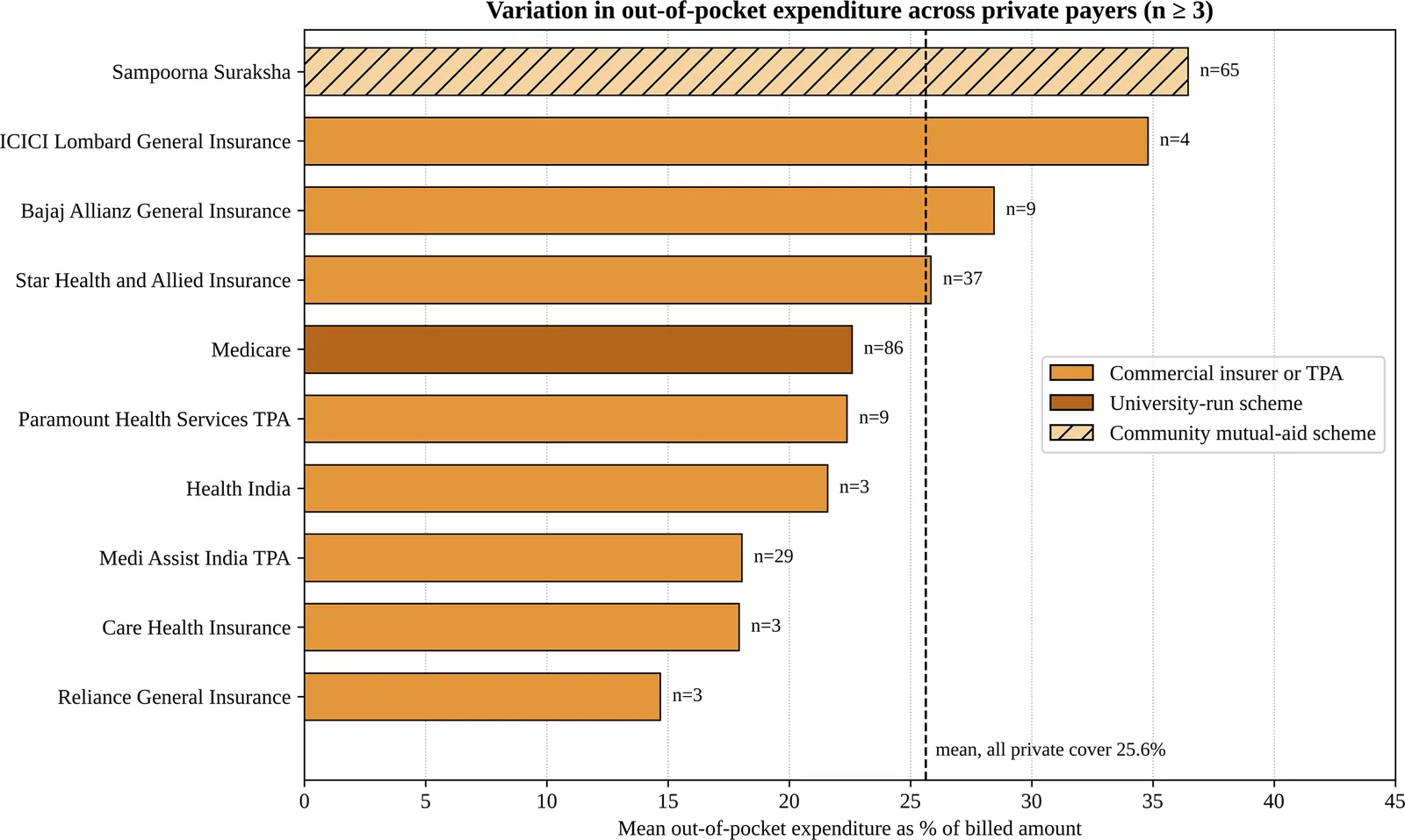
Mean out-of-pocket expenditure as a percentage of billed amount across private payers with at least three patients, shaded by type of payer: commercial insurer or third-party administrator, university-run scheme (Medicare) and community mutual-aid scheme (Sampoorna Suraksha). The dashed line marks the mean across all privately insured patients (25.6%). The twelve payers with fewer than three patients are pooled in supplementary Table S5. Estimates for payers with few patients are exploratory.

**Table 6.** Recorded out-of-pocket expenditure among privately insured patients, by type of payer. TPA, third-party administrator. Medicare is a health scheme operated by the university with which the hospital is affiliated; Sampoorna Suraksha is the community-based mutual-aid scheme of the Shri Kshethra Dharmasthala Rural Development Project. No payer contribution means that nothing was recorded as received from the registered payer. Income cut-off: 10% of Karnataka’s per-capita income (₹33,293). The adjusted difference is from linear regression of OOPE as a percentage of charge on payer type, log charge, age and clinical department, with robust standard errors.

Measure	Commercial insurer or TPA (n = 111)	University-run scheme (n = 86)	Community mutual-aid scheme (n = 65)
Payers, n	20	1	1
Age, years, median (IQR)	36 (29–52)	22 (19–39)	38 (30–45)
Total charge, ₹, median (IQR)	118,304 (62,800–183,267)	68,787 (35,394–134,495)	66,154 (31,981–96,478)
OOPE, ₹, median (95% CI)	13,170 (11,647–16,306)	8,243 (6,216–10,654)	11,410 (8,880–17,567)
OOPE, % of charge: mean (median)	21.7 (15.4)	22.6 (13.8)	36.4 (23.0)
OOPE, % of charge: 90th percentile	43.9	55.2	85.1
Share of charges met by payer, %	77.4	68.0	51.6
Zero OOPE, n	1	1	0
No payer contribution, n	2	1	5
OOPE >40% of charge, n (%)	15 (13.5)	16 (18.6)	18 (27.7)
OOPE >10% of per-capita income, n (%)	24 (21.6)	19 (22.1)	16 (24.6)
Adjusted difference in OOPE, percentage points of charge (95% CI)	Reference	3.3 (−2.8 to 9.4)	14.9 (6.2 to 23.5)

## Discussion

In this cohort, admissions billed at the same tariff ended with very different recorded payments, and the difference followed the arrangement under which the patient paid. Publicly funded insurance was associated with no recorded hospital OOPE for any beneficiary. Private insurance left a partial and unpredictable share with patients. No cover left the whole bill. This ordering—no recorded hospital OOPE under public insurance, a partial share under private insurance, the whole bill under self-payment—is the same ordering we observed among 2,715 COVID-19 inpatients at this institution, where OOPE ran 0%, 21.2% and 100% across the three groups, ^13^ and for obstetric and gynaecological surgery at this centre. ^21^ That the ordering recurs across unrelated clinical contexts at one institution suggests it reflects the financing arrangements more than any particular clinical episode. It does not establish that the arrangements caused it. The main alternative explanation—that differences in OOPE simply track differences in the size of the bill—is weakened by the data, for two reasons. Charges were similar across groups before and after adjustment for age, clinical department and road-user category ( Table 4). And OOPE is measured as a share of each patient’s own bill: a difference in charges of the size the confidence intervals allow cannot generate a gradient from 0% to 100%. The groups were not clinically identical. Uninsured patients were somewhat older, neurosurgical cases were far commoner among them than under AB-PMJAY ( Table 2), and injury severity, length of stay, intensive-care use and procedures were not recorded. These unmeasured differences could still bear on the result, for example through the eligibility of particular cases for cover or the response of payers to them. The findings are therefore associations within one hospital, not causal effects of the financing mechanisms. With that qualification, one point is plain: zero recorded OOPE was delivered, in this hospital, to every beneficiary who reached the scheme. For the uninsured, the gap these data describe is the absence of cover. Whether cover would have closed it for them cannot be tested here.

The most consequential number in this study is not the OOPE gradient but its denominator: four out of five trauma patients (79.7%) were admitted with no cover registered with the hospital, and half of them went on to a recorded hospital bill exceeding a quarter of annual per-capita income. Two explanations are plausible, and they are not mutually exclusive. Some of these patients belong to the ‘missing middle’: insufficiently poor for AB-PMJAY, insufficiently affluent for private cover. ^7^ Others may be AB-PMJAY-eligible but unidentified: in a six-state survey of eligible households, only 62% were aware of the scheme at all, and only about three-quarters of those knew of their own eligibility ^22^—and identification is hardest at precisely the moment of unplanned emergency admission, when patients may carry no documentation and verification competes with acute care. This yields a hypothesis for future research: that some of the 1,274 uninsured patients here were eligible for AB-PMJAY but unidentified, and that eligibility verification in the emergency department would have reduced their OOPE. These records cannot test it, because eligibility was not assessed among the uninsured. Quantifying the eligible-but-unenrolled fraction among self-paying trauma patients is the immediate research priority this study points to.

Similar patterns have been reported in other settings. The most directly comparable injury study, by Prinja and colleagues in North Indian public hospitals, reported CHE in 22.2% of injury-affected households and impoverishment in 12.2% at a 30%-of-consumption threshold. ^12^ The shares here are higher (78.7% of uninsured patients above 10% of per-capita income; 27.9% above 40%), but the two are not comparable: Prinja measured household CHE against consumption in lower-tariff public hospitals, whereas we measure a single admission’s recorded OOPE against average income per person in a private tertiary centre. Both point the same way: injury care leaves uninsured patients with bills that are large by either yardstick. Hospital-based studies elsewhere point the same way where pooled financing for road-trauma care is absent, though at different magnitudes: 67% of households faced catastrophic expenditure in Addis Ababa, and 19% in Ouagadougou, where under 5% of trauma patients held any insurance. ^23^ ^,^ ^24^ Earlier quasi-experimental evaluations of publicly funded insurance in India found similar, though less complete, reductions in OOPE and improvements in access. ^25^ ^,^ ^26^

Among the privately insured, the mean residual payment of 25.6% understates the real failure, which is unpredictability. The median patient paid 16.7% of charges, but the 90th percentile paid 65.5%, nearly one in five paid more than 40%, and 3% had no payment from the registered payer recorded at all, while only 0.8% left with nothing to pay (supplementary Table S6). Some of those with no payer contribution may have been reimbursed after discharge, which hospital records do not show. The unpredictability is not confined to the mutual-aid scheme: among commercial insurers and their administrators alone, the 90th percentile paid 43.9% of charges and 13.5% paid more than 40% ( Table 6). Insurance whose recorded benefit ranges from complete to nil, for the same emergency at the same hospital, fails ex ante as insurance: households cannot know their exposure before the event. Part of that dispersion is structural rather than idiosyncratic: the community mutual-aid scheme, covering self-help-group households, left 14.9 percentage points more of the bill with patients than commercial insurance after adjustment, so voluntary cover performed worst in a scheme designed for self-help-group households. This dispersion, consistent with earlier Indian evidence on incomplete private protection, ^10^ points to benefit design: co-payments, room-rent-linked proportional deductions, sub-limits and consumables exclusions that trauma care triggers unpredictably. Standardising benefit packages, capping deductions for emergency admissions and prospective payment approaches such as diagnosis-related groups could make protection more predictable, which is the property this market currently lacks. ^27^

India has since acted on this gap. In May 2025 the Ministry of Road Transport and Highways notified the Cashless Treatment of Road Accident Victims Scheme under section 162 of the Motor Vehicles Act, 1988; it was launched in February 2026 as the Prime Minister–Road Accident Victims’ Hospitalisation and Assured Treatment (PM-RAHAT) scheme. Any person injured in a motor vehicle crash on any road is entitled to cashless treatment of up to ₹1.5 lakh (₹150,000; about US$1,754) for seven days at a designated hospital, financed from a Motor Vehicle Accident Fund, with no insurance requirement of any kind. ^28^ Entitlement replaces identification: the 1,274 uninsured patients in this cohort would not have needed to be located in a beneficiary database, only recognised as crash victims. Our data indicate how far that entitlement could reach, under explicit assumptions. We assumed that every uninsured admission would have qualified, that the hospital was designated, that every billed component was eligible, and that the scheme would have paid the lesser of the bill and ₹1.5 lakh. The seven-day limit could not be applied, because length of stay was not captured. The estimate is therefore a simplified simulation and an upper bound on what the scheme would have met, not an estimate of its realised effect. On these assumptions, the median uninsured charge of ₹85,091 is 57% of the ceiling, so the median uninsured patient would have been covered in full. But 302 of the 1,274 uninsured patients (23.7%; 95% CI 21.5–26.1) were billed above ₹1.5 lakh, and those who crossed the ceiling crossed it heavily: the part of their bill above the ceiling averaged ₹129,254 (median ₹70,771), close to the value of the cover itself. Across all uninsured admissions the ceiling would have met 73.7% of charges and left 26.3% unmet. This residual is the amount above the ceiling before any other source of financing; what patients would finally have paid could be lower. The share of uninsured patients with OOPE above 10% of per-capita income would fall from 78.7% to 16.8%, above 25% from 50.9% to 11.0%, and above 40% from 27.9% to 7.8% (supplementary Table S7). Because the seven-day limit and the scheme’s other conditions are ignored, the real residual is likely to be larger. PM-RAHAT therefore converts the financing problem in emergency trauma from one of identification into one of adequacy: the entitlement is universal, the ceiling is not. Whether ₹1.5 lakh and seven days are calibrated to the observed cost distribution of trauma admissions is now the empirical question, and hospital billing data of the kind used here are the natural instrument for answering it.

### Strengths and limitations

Strengths include the use of billing records for all eligible admissions with complete billing over a full year at a high-volume centre, a design that holds the institution and tariff constant across payer groups, adjusted comparisons of charges, and payer-level detail rarely reported. The limitations are important. First, this is a single-centre study with only 63 AB-PMJAY beneficiaries. Payer mix and absolute amounts may not generalise to public hospitals or other settings, and zero recorded OOPE under AB-PMJAY at this hospital is not evidence that the scheme protects completely elsewhere. Second, the comparison is observational. Routine records did not capture injury severity, length of stay, intensive-care use or procedures, and the payer groups differed in age and clinical department ( Table 2). Adjustment for the observed differences changed little, but residual confounding by severity, eligibility or payer behaviour cannot be excluded, so the findings are associations, not causal effects. Third, OOPE is the patient payment recorded in hospital billing. Medicines, diagnostics or consumables bought outside the hospital, transport, follow-up care and indirect costs such as lost income and caregiving were not captured, so the total burden is underestimated in every group, AB-PMJAY included. Reimbursements received after discharge are also invisible, so recorded OOPE may overstate the final burden of some privately insured patients, and of any insured patients who paid the hospital themselves and were therefore recorded as self-paying. Fourth, total charges are gross amounts at the hospital tariff; for AB-PMJAY, the amount the State Health Agency actually reimbursed at package rates was not available. Fifth, household consumption was not measured, so the income-threshold indicator cannot identify household catastrophe or impoverishment and is not comparable with survey-based CHE. Sixth, the analysed set covers inpatient admissions only. Patients who died before admission, were treated without admission, or were taken to other hospitals are absent, as are the 102 admissions under other public or employer schemes and the 10 with incomplete billing. The payer mix therefore describes admitted patients financed through the three study modes, not all crash victims. The direction of the resulting bias is uncertain: deaths before admission remove the most severe injuries from every group, while patients unable to reach or afford a tertiary centre are likely to be poorer and uninsured, so the burden of the uninsured may be understated. Seventh, the privately insured group combines commercial insurance, a university-run scheme and a community mutual-aid scheme; its mean averages over different benefit designs, and estimates for small payers are exploratory. Eighth, road-user category was recorded as other or unspecified in 36.2% of admissions (579 of 1,599), largely because the external-cause code frequently records only that a collision occurred between two vehicle types without stating which the patient occupied; we draw no detailed conclusions from it. Finally, the study period precedes the national cashless trauma entitlement, so the payer distribution reported here describes the position immediately before that entitlement took effect rather than the position today. These constraints motivate multi-site, prospectively linked studies incorporating clinical severity and household economic data.

## Conclusions

Among road traffic accident admissions at one tertiary hospital, billed under a single tariff, recorded out-of-pocket expenditure differed sharply by payer while charges did not. No AB-PMJAY beneficiary had any recorded hospital OOPE, private insurance left a partial and inconsistent share with patients, and uninsured patients—the large majority—paid the full bill. These are associations within one hospital, and they concern the hospital bill alone. These data describe the position immediately before a universal cashless entitlement was extended to every road crash victim in India. ^28^ They also suggest its limit: nearly a quarter of the uninsured patients here (23.7%) were billed above the scheme’s ₹1.5 lakh ceiling, by a mean of ₹129,254. Calibrating that ceiling against the observed cost distribution of trauma admissions, and standardising private insurance benefit design, are steps worth testing toward more equitable financial protection for trauma patients in India.

## Ethics and consent

Ethical approval for this study was obtained from the Institutional Ethics Committee – 2 (Student Research), Kasturba Medical College and Kasturba Hospital, Manipal (reference IEC2: 703/2024; DHR registration EC/NEW/INST/2024/KA/0509), approved on 30 December 2024. Permission to access patient records held by the Medical Records Department was granted by the Medical Superintendent of Kasturba Hospital, who is the custodian of those records; that permission was submitted to the Institutional Ethics Committee as part of the approval process. Individual informed consent was not required, as the study was a retrospective analysis of de-identified, routinely collected billing and admission records involving no patient contact and no intervention. The study was conducted in accordance with the Declaration of Helsinki.

## Data Availability

Figshare: Same hospital, same tariff, different protection: out-of-pocket expenditure among road traffic accident admissions under public insurance, private insurance and self-payment in India. https://doi.org/10.6084/m9.figshare.33183602.v3 ^29^ This project contains the following underlying data: RTA_OOPE_patient_level.xlsx. Patient-level billing and admission records for all 1,599 road traffic accident admissions analysed. The file contains, for each admission, a sequential study identifier, payer group, insurer or third-party administrator name where applicable, age in years, clinical department and hospital department code, ICD-10 external cause description, road-user category, discharge outcome and discharge code, total billed amount, amount met by the payer, out-of-pocket expenditure, and out-of-pocket expenditure as a percentage of the billed amount. A codebook giving variable definitions and permitted values is included in the same file. The data are anonymised: hospital account numbers, patient names, all calendar dates, addresses and contact details have been removed, and no personal identifying information is included. Figshare: Same hospital, same tariff, different protection: out-of-pocket expenditure among road traffic accident admissions under public insurance, private insurance and self-payment in India. https://doi.org/10.6084/m9.figshare.33183602.v3 ^29^ This project contains the following extended data: Supplementary_tables_S1_S10.xlsx. Table S1, study flow and exclusions. Table S2, baseline characteristics by payer status. Table S3, total charges and recorded out-of-pocket expenditure by payer, with 95% confidence intervals. Table S4, recorded out-of-pocket expenditure above 10%, 25% and 40% of per-capita income, with cut-offs and 95% confidence intervals. Table S5, out-of-pocket expenditure by private payer and type of payer. Table S6, distribution of realised benefit among privately insured patients. Table S7, simplified simulation of the 2025 cashless trauma entitlement ceiling applied to the uninsured group, with its assumptions. Table S8, unadjusted and adjusted comparisons of total charges. Table S9, total charges by clinical department and payer group. Table S10, recorded out-of-pocket expenditure by type of private payer. STROBE_checklist.docx. Completed STROBE checklist for cross-sectional studies. RTA_OOPE_analysis.py. Python script that reproduces every estimate in the article from the underlying data, with a fixed random seed. Data are available under the terms of the Creative Commons Attribution 4.0 International license (CC-BY 4.0).
