# Supplementary material for: Same Hospital, Same Tariff, Different Protection: Out-of-pocket Expenditure among Road Traffic Accident Admissions under Public Insurance, Private Insurance and Self-payment in India
Source: F1000Res. 2026 Nov 6;15:1690. Originally published 2026 Oct 8. [Version 2] doi: 10.12688/f1000research.188694.2 (PMC13770162; doi:10.12688/f1000research.188694.2)
Supplement: Peer review history

# Reviewer report: Reviewer response for version 2

**DOI:** 10.5256/f1000research.214011.r538954

**Published:** 2026-11-14

**Authors:** Pushpendra Singh

**Affiliations:** 1 Somaiya Vidyavihar University, mumbai, Maharashtra, India

The authors have satisfactorily addressed all the comments.

Is the work clearly and accurately presented and does it cite the current literature?

Yes

If applicable, is the statistical analysis and its interpretation appropriate?

No

Are all the source data underlying the results available to ensure full reproducibility?

Yes

Is the study design appropriate and is the work technically sound?

No

Are the conclusions drawn adequately supported by the results?

Partly

Are sufficient details of methods and analysis provided to allow replication by others?

Partly

Reviewer Expertise:

Health Economics (Insurance and Financing)

I confirm that I have read this submission and believe that I have an appropriate level of expertise to confirm that it is of an acceptable scientific standard.

# Reviewer report: Reviewer response for version 2

**DOI:** 10.5256/f1000research.214011.r538953

**Published:** 2026-11-10

**Authors:** Mohammed Shoyaib Khazi

**Affiliations:** 1 Navodaya Medical College, Raichur, Karnataka, India

The authors have made necessary changes and have adequately addressed all the comments given. Recommend for Acceptance.

Is the work clearly and accurately presented and does it cite the current literature?

Partly

If applicable, is the statistical analysis and its interpretation appropriate?

Yes

Are all the source data underlying the results available to ensure full reproducibility?

Yes

Is the study design appropriate and is the work technically sound?

Yes

Are the conclusions drawn adequately supported by the results?

Partly

Are sufficient details of methods and analysis provided to allow replication by others?

Partly

Reviewer Expertise:

Community Medicine

I confirm that I have read this submission and believe that I have an appropriate level of expertise to confirm that it is of an acceptable scientific standard.

# Reviewer report: Reviewer response for version 1

**DOI:** 10.5256/f1000research.208259.r528545

**Published:** 2026-10-24

**Authors:** Pushpendra Singh

**Affiliations:** 1 Somaiya Vidyavihar University, mumbai, Maharashtra, India

 I started reading this manuscript with deep interest, but came across the following issues that need to be addressed 

 1. The study uses Karnataka’s average per-capita income instead of patients’ household income or consumption. This applies the same financial threshold to rich and poor households and cannot establish which households experienced catastrophic spending.

 2. Neurosurgical admissions account for 3.2% of AB-PMJAY patients but 32.8% of uninsured patients. Such differences weaken the claim that insurance alone explains the results.

 3. Injury severity, length of stay, ICU use, and treatment procedures are unavailable. Without these variables, the study cannot establish that patients received “identical care.”

 4. The reported p-value of 0.70 means that the analysis did not detect a difference; it does not establish that treatment charges were equivalent across groups.

 5. The analysis does not account even for observed differences in age and clinical department. Adjusted analysis could improve the comparison, although missing clinical and household information would still prevent strong causal conclusions.

 6. The records do not establish whether patients paid for medicines or services elsewhere, transport, or follow-up care. The finding should therefore be described as zero recorded hospital OOPE among 63 AB-PMJAY beneficiaries.

 7. Private insurance is defined partly through direct settlement with the hospital, yet some privately insured patients received no benefit. Explain how rejected claims, later reimbursements, and insured patients initially paying themselves were classified.

 8. Results from one hospital, including only 63 AB-PMJAY beneficiaries, cannot establish that public insurance provides complete protection generally or that lack of coverage is the only problem.

 9. The paper could offer a useful descriptive comparison of hospital payments, but its claims about causal effects, household financial protection, and policy effectiveness require substantial revision.

Is the work clearly and accurately presented and does it cite the current literature?

Yes

If applicable, is the statistical analysis and its interpretation appropriate?

No

Are all the source data underlying the results available to ensure full reproducibility?

Yes

Is the study design appropriate and is the work technically sound?

No

Are the conclusions drawn adequately supported by the results?

Partly

Are sufficient details of methods and analysis provided to allow replication by others?

Partly

Reviewer Expertise:

Health Economics (Insurance and Financing)

I confirm that I have read this submission and believe that I have an appropriate level of expertise to confirm that it is of an acceptable scientific standard, however I have significant reservations, as outlined above.

# Response

**Published:** 2026-10-31

**Authors:** rajesh kamath

**Affiliations:** Prasanna School of Public Heal, Manipal Academy of Higher Education, Manipal, Karnataka, India

Dear Dr Singh,

 Thank you for your careful reading and for raising points that go to the heart of what the study can and cannot show. We agree with all nine. The revision now treats the paper as a descriptive comparison of hospital payments within one hospital, with adjusted analyses and stated limits. The title now reads “Same hospital, same tariff, different protection: out-of-pocket expenditure among road traffic accident admissions under public insurance, private insurance and self-payment in India”. Our responses follow point by point. Locations refer to section headings and tables of Version 2.

***1.*** *The study uses Karnataka’s average per-capita income instead of patients’ household income or consumption … cannot establish which households experienced catastrophic spending.*

 You are right, and we no longer claim that it does. The term “catastrophic health expenditure” has been dropped for our estimates. They are now described as an admission-level indicator of potential financial hardship: recorded OOPE above 10%, 25% and 40% of per-capita income, with the rupee cut-offs stated (₹33,293, ₹83,232 and ₹133,170). Methods now says what the indicator cannot do. It applies one denominator to every patient, it does not distinguish richer households from poorer ones, it compares one admission with a year’s income per person rather than per household, and it cannot identify households that experienced catastrophe or impoverishment.

**Change made: **Methods, OOPE relative to per-capita income; Results; Table 5; Figure 3; Strengths and limitations (fifth limitation).

***2.*** *Neurosurgical admissions account for 3.2% of AB-PMJAY patients but 32.8% of uninsured patients. Such differences weaken the claim that insurance alone explains the results.*

 Thank you; this is an important observation, and we have examined it directly. Two findings are relevant. First, the excess of neurosurgical admissions did not inflate uninsured bills. Among uninsured patients, the median neurosurgical charge (₹51,016) was less than half the median orthopaedic charge (₹109,016). Second, adjustment for clinical department, age and road-user category changed the comparison of charges little (adjusted ratio of medians against the uninsured 0.95, 95% CI 0.85–1.08, for AB-PMJAY). Within orthopaedics, the largest department, median charges were ₹111,097, ₹117,323 and ₹109,016 (p=0.56). We also report a sensitivity analysis that points the other way: with neurosurgery excluded, mean AB-PMJAY charges were 20% lower after adjustment (ratio of means 0.80, 0.69–0.92).

 We agree that insurance alone cannot be said to explain the results, and statements to that effect have been removed. One observation remains relevant. OOPE is measured as a share of each patient’s own bill, so differences in charges of the size these data allow cannot produce a gradient from 0% through 25.6% to 100% of the bill. The difference in OOPE is therefore not an artefact of different bills, but it remains an association, and the Discussion says so.

**Change made: **Results, Total charges were similar across payer groups, before and after adjustment; Table 4; Tables S8 and S9; Discussion, first paragraph.

***3.*** *Injury severity, length of stay, ICU use, and treatment procedures are unavailable. Without these variables, the study cannot establish that patients received “identical care.”*

 Agreed. “Identical care”, “identical prices” and “identical institutional conditions” have been removed throughout. The claim is now limited to what the data show: the groups were billed under the same tariff at the same hospital. The missing clinical variables are named in the Discussion and limitations as a source of possible residual confounding.

**Change made: **Title; Abstract; Introduction, final paragraph; Discussion, first paragraph; Strengths and limitations (second limitation); Conclusions.

***4.*** *The reported p-value of 0.70 means that the analysis did not detect a difference; it does not establish that treatment charges were equivalent across groups.*

 You are right, and the text no longer says or implies that it does. A sentence in Methods now makes the point, with a reference. In place of the claim, we report ratios and differences of medians with 95% confidence intervals, unadjusted and adjusted (Table 4). The text states the range of differences these intervals leave open: after adjustment, median AB-PMJAY charges between 15% lower and 8% higher than uninsured charges. We did not specify an equivalence margin in advance, so we have not presented a formal equivalence test.

**Change made: **Methods, Statistical analysis; Results, Total charges; Table 4.

***5.*** *The analysis does not account even for observed differences in age and clinical department …*

 Thank you; this has been added. Charges were compared after adjustment for age, clinical department and road-user category, by median regression of log charges and by a gamma model with robust standard errors. Sensitivity analyses used finer hospital units and excluded neurosurgery (Table 4; Table S8). For the income thresholds, adjusted risk ratios for private insurance against no cover were 0.28, 0.19 and 0.21 at the three thresholds. We agree that missing clinical and household information still rules out strong causal conclusions, and the revision draws none.

**Change made: **Methods, Statistical analysis; Results; Tables 4 and S8.

***6.*** *The records do not establish whether patients paid for medicines or services elsewhere, transport, or follow-up care. The finding should therefore be described as zero recorded hospital OOPE among 63 AB-PMJAY beneficiaries.*

 Agreed, and we have adopted your wording. The outcome is now defined as recorded hospital OOPE, and Methods lists what the records do not capture. The AB-PMJAY finding is reported as zero recorded hospital OOPE for all 63 beneficiaries (95% CI 94.3–100%). The text adds that, with 63 beneficiaries, the data cannot exclude non-zero recorded OOPE in up to about 6% of AB-PMJAY admissions at this hospital, and that they say nothing about payments outside the hospital bill.

**Change made: **Methods, Variables and outcome measures; Results, Zero recorded hospital out-of-pocket expenditure; Strengths and limitations (third limitation).

***7.*** *Private insurance is defined partly through direct settlement with the hospital, yet some privately insured patients received no benefit. Explain how rejected claims, later reimbursements, and insured patients initially paying themselves were classified.*

 Thank you for spotting this; the Version 1 definition was indeed inconsistent with the data. Payer group follows the payer recorded against the admission in the hospital billing system. Private insurance is now defined as any non-government arrangement, registered with the hospital for the admission, under which a third-party payer was liable to settle the bill directly with the hospital. Admissions stay in this group whatever the outcome of the claim. Eight of the 262 privately insured admissions had nothing recorded as received from the registered payer, and for these the recorded patient payment equals the full charge. This happens when a claim is rejected, or when the patient settles the bill and claims from the payer directly. The hospital records do not distinguish the two.

 Any reimbursement a patient received after discharge is not visible in hospital records. Patients who registered no cover with the hospital were recorded as self-paying, so an insured patient who paid the hospital and claimed later without registering the policy would appear in the uninsured group. Both possibilities are now acknowledged as limitations: recorded OOPE may overstate the final burden of some privately insured patients and of any such patients in the uninsured group. Five of the eight admissions with no payer contribution were members of the community mutual-aid scheme.

**Change made: **Methods, Participants and eligibility; Results, Variation across private payers; Table 6; Table S6; Discussion, fourth paragraph; Strengths and limitations (third limitation).

***8.*** *Results from one hospital, including only 63 AB-PMJAY beneficiaries, cannot establish that public insurance provides complete protection generally or that lack of coverage is the only problem.*

 Agreed. The first limitation now says that zero recorded OOPE under AB-PMJAY at this hospital is not evidence that the scheme protects completely elsewhere, and the 95% confidence interval for that proportion is reported. The statement that the binding failure was “the absence of cover, not its design” has been removed. The Discussion now says only that, for the uninsured, the gap these data describe is the absence of cover, and that whether cover would have closed it cannot be tested here.

**Change made: **Results, Zero recorded hospital OOPE; Discussion, first paragraph; Strengths and limitations (first limitation); Conclusions.

***9.*** *The paper could offer a useful descriptive comparison of hospital payments, but its claims about causal effects, household financial protection, and policy effectiveness require substantial revision.*

 Thank you; we think this is the right framing, and the revision follows it. Claims of causal effect are withdrawn and the findings are presented as associations within one hospital. Claims about household financial protection are withdrawn, and the income-threshold indicator is described as an admission-level measure. Policy implications are framed as hypotheses and steps to test. The PM-RAHAT analysis is presented as a simplified simulation with explicit assumptions, an upper bound rather than an estimate of the scheme’s effect.

**Change made: **Throughout; summarised in the Update Text.

 We are grateful for your report, which has sharpened the paper considerably.

 Warm regards,

 Rajesh

# Reviewer report: Reviewer response for version 1

**DOI:** 10.5256/f1000research.208259.r528553

**Published:** 2026-10-14

**Authors:** Mohammed Shoyaib Khazi

**Affiliations:** 1 Navodaya Medical College, Raichur, Karnataka, India

The manuscript addresses an important question concerning financial risk protection during emergency trauma care in India. The use of patient-level billing records from a single tertiary hospital is a notable strength because it reduces some of the between-hospital variation that complicates comparisons of out-of-pocket expenditure (OOPE). The sample size is reasonable, and the analysis of variation within the privately insured group could be informative for health-financing policy. However, several aspects of the methodology and interpretation require clarification. In particular, the manuscript occasionally draws stronger causal and policy conclusions than the retrospective observational design supports. The authors are encouraged to address the following major concerns.

**Major comments**

**1. Clarify what is meant by identical prices and comparable treatment costs.**

 The central argument is that treatment charges were similar across payer groups and that differences in OOPE can therefore be attributed to the payment mechanism. However, a non-significant Kruskal–Wallis test (p = 0.70) does not establish equivalence of charges. Furthermore, the payer groups differed in age and clinical department, particularly in the proportion of neurosurgical admissions. Injury severity, length of stay, intensive care utilisation and procedures were unavailable.

 The authors should clarify whether total charges represent the gross amount billed before payer-specific adjustments, the amount approved for reimbursement, or the final amount considered payable. This distinction is particularly important when comparing a publicly funded scheme with private insurance and self-payment.

 Please avoid concluding that differences in OOPE are attributable to the payment mechanism *alone*. The findings demonstrate a strong association between payer status and patient payments in this hospital, but they do not establish that all other relevant determinants have been eliminated. The interpretation should acknowledge the residual differences in case mix and the possibility of payer-specific reimbursement arrangements.

**2. Reconsider the definition and interpretation of catastrophic health expenditure.**

 The study compares a single hospital admission's OOPE against 10%, 25% and 40% of Karnataka's annual per-capita income. Although the authors acknowledge that this is a proxy, the resulting estimates are repeatedly described as catastrophic health expenditure.

 Conventional CHE measures generally use household consumption expenditure or capacity to pay as the denominator. Annual per-capita income is not equivalent to either measure, and comparing an individual admission's expenditure with a fraction of annual per-capita income does not establish whether the household experienced financial catastrophe or impoverishment.

 The authors should explain the rationale for selecting these thresholds, provide the corresponding monetary cut-offs, and consistently distinguish the resulting measure from conventional household-level CHE. If household-level data are unavailable, consider describing the outcome as an income-threshold-based indicator of potential financial hardship. The abstract, results, discussion and conclusions should avoid presenting these estimates as directly comparable with nationally representative CHE estimates or studies using household consumption expenditure.

**3. Clarify the calculation of OOPE under AB-PMJAY and private insurance.**

 The finding that all 63 AB-PMJAY beneficiaries incurred zero OOPE is important. However, the manuscript states that cashless treatment implied an expected OOPE of zero, which risks making the outcome partly dependent on the definition used.

 Please explain precisely how patient payments were identified and whether the records captured all payments made outside the hospital billing system, including payments for medicines, diagnostics, consumables or services obtained elsewhere. Also clarify whether the amount met by AB-PMJAY was based on the actual claim settlement, the approved package amount or another administrative measure.

 For private insurance, please describe how reimbursements, deductions, co-payments, exclusions and amounts not approved by insurers were distinguished. If only hospital-recorded payments were available, this limitation should be explicitly acknowledged. The authors should distinguish zero recorded hospital OOPE from complete financial protection against all costs associated with trauma care.

**4. Address the heterogeneity of the private insurance category.**

 The private insurance group combines commercial indemnity insurance, third-party administrators and a community-based mutual-aid scheme, Sampoorna Suraksha. These arrangements may differ substantially in their financing structures, benefit packages, eligibility criteria and reimbursement procedures.

 The finding that this group incurred a mean OOPE equivalent to 25.6% of charges therefore represents an average across heterogeneous financing arrangements rather than the performance of conventional private health insurance alone.

 The authors should justify the decision to combine these categories, clearly distinguish insurance products from third-party administrators and mutual-aid arrangements, and interpret the aggregate estimate accordingly. Where sample sizes permit, separate descriptive results for commercial insurance and community-based arrangements would strengthen the analysis. The insurer-level results should be treated as exploratory, particularly for payers represented by small numbers of patients.

**5. Strengthen the statistical interpretation of differences between payer groups.**

 The study reports unadjusted comparisons using non-parametric tests. Although this approach may be appropriate for describing skewed expenditure distributions, it does not account for differences in patient characteristics and clinical presentation.

 The authors should consider whether an adjusted analysis is feasible using the available variables, such as age, clinical department and road-user category. Given the absence of injury severity and resource-utilisation data, any adjusted results would remain limited and should not be interpreted causally. If adjustment is not feasible or appropriate, the manuscript should explain why and frame the comparisons as descriptive.

 Additionally, the assertion that total charges were equivalent should be moderated unless an equivalence framework with a prespecified margin is used. Please report confidence intervals consistently for the main expenditure estimates and provide sufficient information to assess the magnitude and precision of the observed differences, rather than relying predominantly on p-values.

**6. Reassess the counterfactual analysis of the PM-RAHAT scheme.**

 The analysis applying the ₹1.5 lakh ceiling to the previously uninsured patients is potentially valuable for policy planning. However, it assumes that the scheme would cover the relevant hospital charges up to the ceiling and that the historical admissions would have qualified for the same level of support.

 Please clarify the assumptions used to estimate the proportion of charges covered and the residual liability. In particular, explain whether coverage is assumed to equal the lesser of the hospital bill and ₹1.5 lakh, and how the seven-day treatment limit, eligible treatment components, designated-hospital requirements and other scheme conditions were handled.

 The available billing data cannot establish what patients would actually have received under the scheme. The estimates should therefore be presented as a simplified financial simulation under specified assumptions, not as a direct estimate of the scheme's realised effectiveness. The authors should also distinguish the amount of the bill exceeding the ceiling from the amount that would remain payable by patients after accounting for any other available financing.

**7. Moderate the causal language and policy conclusions.**

 Statements such as “the financial fate of a trauma patient ... was decided by a single factor” and “the binding failure ... was the absence of cover, not its design” are stronger than the study design supports. The analysis identifies substantial differences in recorded OOPE by payer status, but it does not fully account for differences in clinical need, household resources, insurance eligibility, benefit design or access to care.

 Similarly, the study cannot establish that on-site eligibility verification would have eliminated OOPE for a particular proportion of uninsured patients, because eligibility was not assessed among those patients. This should be framed as a plausible hypothesis for future research rather than a demonstrated intervention effect.

 Please revise the abstract and discussion to distinguish observed findings from inferred mechanisms and policy implications. The policy relevance can be retained without implying causal identification.

**8. Clarify the study population and potential selection bias.**

 The analysis includes patients admitted through the emergency department and excludes patients who died before inpatient admission. It also excludes 102 patients financed through other arrangements and 10 privately insured patients with incomplete billing records.

 Please discuss how these exclusions may affect the payer distribution and observed financial burden. In particular, the results may not represent all road traffic injury patients, including those treated without admission, those unable to reach this hospital, or those whose injuries resulted in death before admission. The title and conclusions should consistently refer to admitted patients or the study cohort rather than implying coverage of all emergency trauma patients in India.

 A clearer description of the selection process and the implications of excluding other financing arrangements would improve transparency and generalisability.

**Minor comments**

1. **Study design and reporting:** Please reconsider whether describing this as a retrospective observational study of hospital admissions is more precise than reporting STROBE for cross-sectional studies. Ensure the selected reporting checklist matches the study's design and clarify the unit of analysis.
2. **Confidence intervals:** The manuscript provides bootstrap confidence intervals for some medians but not consistently for all payer groups. Please report uncertainty estimates consistently where feasible and explain the bootstrap procedure, including the resampling unit.
3. **Interpretation of zero OOPE:** Use “zero recorded OOPE” where appropriate unless the data establish that patients incurred no direct payments outside the hospital billing system.
4. **Terminology:** Use “uninsured/self-paying” consistently and clarify that this category refers to patients without one of the three specified financing arrangements, rather than necessarily establishing that they had no access to any other form of assistance.
5. **Road-user classification:** Given that 36.2% of cases were classified as other or unspecified, acknowledge that the available ICD-10 codes provide limited information on the patient's precise road-user role. Avoid drawing detailed conclusions from this variable.
6. **Presentation of results:** Consider adding a concise table showing the median OOPE, proportion with zero recorded OOPE, and proportion exceeding each income-based threshold, together with denominators and confidence intervals where appropriate. Clearly distinguish mean OOPE as a percentage of charges from the median percentage.
7. **Reproducibility:** The availability of de-identified patient-level data is a strength. Please ensure that the supplementary tables, variable definitions and reported figures can be reconciled with the underlying dataset, particularly the insurer-level summaries and PM-RAHAT counterfactual estimates.

**Conclusion**

 This is a relevant study with potentially useful empirical findings on financial protection during emergency trauma care. The common-hospital setting and detailed billing records are important strengths. However, the interpretation of payer differences, the operationalisation of CHE, and the assumptions underlying the policy simulation need greater methodological precision. Addressing these issues would substantially improve the manuscript's credibility and usefulness for health-financing policy.

Is the work clearly and accurately presented and does it cite the current literature?

Partly

If applicable, is the statistical analysis and its interpretation appropriate?

Yes

Are all the source data underlying the results available to ensure full reproducibility?

Yes

Is the study design appropriate and is the work technically sound?

Yes

Are the conclusions drawn adequately supported by the results?

Partly

Are sufficient details of methods and analysis provided to allow replication by others?

Partly

Reviewer Expertise:

Community Medicine

I confirm that I have read this submission and believe that I have an appropriate level of expertise to confirm that it is of an acceptable scientific standard, however I have significant reservations, as outlined above.

# Response

**Published:** 2026-10-31

**Authors:** rajesh kamath

**Affiliations:** Prasanna School of Public Heal, Manipal Academy of Higher Education, Manipal, Karnataka, India

Dear Dr. Khazi,

 Thank you for reading the manuscript so closely, and for a report that has made it a better paper. We have taken up almost all of your suggestions. In brief: charges are now compared with and without adjustment for case-mix, with confidence intervals in place of the claim of equivalence; the outcome is described precisely as recorded hospital OOPE; “catastrophic health expenditure” has been replaced by an explicitly admission-level income-threshold indicator; the private insurance group is broken down by type of payer; the PM-RAHAT simulation now states its assumptions; and the causal language has been moderated throughout, including in the title, which now reads “Same hospital, same tariff, different protection: out-of-pocket expenditure among road traffic accident admissions under public insurance, private insurance and self-payment in India”. Our responses follow point by point. Locations refer to section headings and tables of Version 2, as page numbers change on typesetting.

**Major comments**

***1. Identical prices and comparable treatment costs.*** *The central argument is that treatment charges were similar across payer groups … Please avoid concluding that differences in OOPE are attributable to the payment mechanism alone.*

 Thank you. We agree with each part of this comment. On what “total charge” means: it is the gross amount billed for the admission at the hospital’s standard tariff, before any payer-specific settlement, deduction or discount, and the same tariff applied to all three groups. It is not the amount approved for reimbursement or the final amount payable. For AB-PMJAY admissions, the finance extract recorded the gross bill and the patient’s payment, and the scheme’s share is their difference. The amount actually reimbursed to the hospital by the State Health Agency at the scheme’s package rates was not part of the extract. We now say so, both in Methods and as a limitation.

 On equivalence: you are right that p=0.70 does not establish it. We have removed that claim and now report the size and precision of the differences instead (point 5 below). On attribution: the statement that differences in OOPE are attributable to the payment mechanism alone has been removed, here and wherever else it appeared. The Discussion now presents the findings as associations within one hospital. It acknowledges the residual differences in case-mix, the unrecorded measures of severity, and the possibility that payers responded differently to particular cases.

**Change made: **Methods, Variables and outcome measures (definition of total charge; how the AB-PMJAY amount was derived; what the extract does not contain); Results, Total charges were similar across payer groups, before and after adjustment; Table 4; Discussion, first paragraph; Strengths and limitations (second and fourth limitations).

***2. Definition and interpretation of catastrophic health expenditure.*** *Conventional CHE measures generally use household consumption expenditure or capacity to pay as the denominator … consider describing the outcome as an income-threshold-based indicator of potential financial hardship.*

 We accept this point fully, and have adopted the term you suggest. The estimates are no longer called catastrophic health expenditure. The Methods section, now titled “OOPE relative to per-capita income”, describes them as an admission-level indicator of potential financial hardship. It explains the thresholds: 10% and 25% were the thresholds of SDG indicator 3.8.2 against total household expenditure or income, and 40% is the conventional capacity-to-pay threshold, which the Inter-Agency and Expert Group on SDG Indicators proposed for the indicator in its 2025 comprehensive review, applied to the household’s discretionary budget. It gives the monetary cut-offs: ₹33,293, ₹83,232 and ₹133,170. It states that the indicator cannot establish household catastrophe or impoverishment and is not comparable with survey-based CHE. The abstract, Results, Discussion, Figure 3 and Tables 5 and S4 use the new terminology. The comparison with Prinja et al. now says plainly that the two measures are not comparable.

**Change made: **Methods, OOPE relative to per-capita income; Results, OOPE relative to per-capita income; new Table 5; revised Figure 3 and legend; Discussion, third paragraph; Strengths and limitations (fifth limitation); two new references (25, 26).

***3. Calculation of OOPE under AB-PMJAY and private insurance.*** *The manuscript states that cashless treatment implied an expected OOPE of zero, which risks making the outcome partly dependent on the definition used …*

 Thank you. That sentence was poorly chosen, and it has been removed. OOPE was observed, not assumed. For every admission, AB-PMJAY admissions included, it is the patient payment recorded in the hospital billing system after any payment by the registered payer; all 63 AB-PMJAY admissions had a recorded patient payment of zero. The AB-PMJAY amount in the dataset is the gross bill less that recorded payment. It is not the claim settlement or the approved package amount, neither of which was in the extract.

 For private insurance, the records give the gross bill, the amount settled by the payer and the patient’s payment. They do not itemise deductions, co-payments, exclusions or disallowed amounts, so we cannot separate these components, and we say so. How rejected claims and later reimbursements were classified is now described in Methods (please see also our response to Reviewer 2, point 7). The records do not capture medicines, diagnostics or consumables bought elsewhere, transport or follow-up care. The outcome is therefore called “recorded hospital OOPE” throughout, and the text now distinguishes zero recorded hospital OOPE from complete financial protection.

**Change made: **Methods, Participants and eligibility and Variables and outcome measures; Results, Zero recorded hospital out-of-pocket expenditure; Strengths and limitations (third limitation); Data sources wording (“amounts settled by payers”).

***4. Heterogeneity of the private insurance category.*** *The private insurance group combines commercial indemnity insurance, third-party administrators and a community-based mutual-aid scheme …*

 We agree, and on revisiting the payers we found that the category has three components rather than two. They are commercial insurers and their third-party administrators (n=111, 20 payers); Medicare, a health scheme operated by the university with which the hospital is affiliated (n=86); and Sampoorna Suraksha, the community mutual-aid scheme (n=65). Methods now describes each, and gives the reason for keeping them in one group:  all three are non-government arrangements under which a third party settles the bill directly with the hospital. That is the mode of financing the study contrasts with AB-PMJAY and with self-payment, and keeping the group intact also preserves the comparison reported in Version 1.

 Results are now reported separately by type (new Table 6; Table S10). Mean OOPE was 21.7% of charges under commercial cover, 22.6% under the university-run scheme and 36.4% under the mutual-aid scheme. After adjustment for charge, age and clinical department, members of the mutual-aid scheme paid 14.9 percentage points more of their bill than patients with commercial cover (95% CI 6.2–23.5); the university-run scheme did not differ from commercial cover (3.3 points; −2.8 to 9.4). The Discussion now reads the aggregate 25.6% as an average across these designs. It also notes that the unpredictability is not confined to the mutual-aid scheme: within commercial cover alone, the 90th percentile paid 43.9% of charges. Payer-level estimates for payers with few patients are described as exploratory.

**Change made: **Methods, Participants and eligibility; Results, Variation across private payers; new Table 6; Figure 4 (now shaded by type of payer) and legend; Table S5 (type of payer added); Table S10; Discussion, fourth paragraph; Strengths and limitations (seventh limitation).

***5. Statistical interpretation of differences between payer groups.*** *The authors should consider whether an adjusted analysis is feasible using the available variables, such as age, clinical department and road-user category … the assertion that total charges were equivalent should be moderated unless an equivalence framework with a prespecified margin is used.*

 Thank you. We have done both. Total charges were modelled on payer group with adjustment for age (linear and quadratic terms), clinical department and road-user category, in two ways. The first is a median regression of log charges, which gives adjusted ratios of medians: 0.95 (95% CI 0.85–1.08) for AB-PMJAY and 1.02 (0.89–1.14) for private insurance, against the uninsured. The second is a gamma model with log link and robust standard errors, which gives adjusted ratios of means: 0.86 (0.71–1.03) and 0.99 (0.86–1.15). Two sensitivity analyses used seven hospital-unit categories in place of four departments, and excluded neurosurgical admissions. Charges were also compared within each clinical department (Table 4; Tables S8 and S9). One result points the other way, and we report it: with neurosurgery excluded, mean AB-PMJAY charges were 20% lower than uninsured charges after adjustment (0.80, 0.69–0.92), although medians did not differ. For the income thresholds, privately insured patients had adjusted risk ratios of 0.28 (0.22–0.35), 0.19 (0.13–0.27) and 0.21 (0.13–0.35) relative to the uninsured at the 10%, 25% and 40% thresholds. AB-PMJAY could not be modelled, as no beneficiary crossed any threshold. All of these are presented as associations.

 On equivalence: we did not specify an equivalence margin before the analysis, and a margin chosen after seeing the data would not give a valid equivalence test. We have therefore removed the claim of equivalence. The text now states the range of differences that the confidence intervals leave open: after adjustment, median AB-PMJAY charges between 15% lower and 8% higher than uninsured charges, and median private charges between 11% lower and 14% higher. Confidence intervals are now reported consistently for the main estimates (Tables 4–6; Tables S3, S4 and S10).

**Change made: **Methods, Statistical analysis; Results, Total charges were similar across payer groups, before and after adjustment, and OOPE relative to per-capita income; new Tables 4 and 5; Tables S8 and S9; three new references (27–29).

***6. Counterfactual analysis of the PM-RAHAT scheme.*** *Please clarify the assumptions used to estimate the proportion of charges covered and the residual liability …*

 We agree, and the paragraph has been rewritten as a simplified simulation with its assumptions stated. We assumed that every uninsured admission would have qualified, that the hospital was designated, that every billed component was eligible, and that the scheme pays the lesser of the bill and ₹1.5 lakh. The seven-day limit could not be applied, because length of stay was not captured. The text now says that the estimate is an upper bound on what the scheme would have met, not an estimate of its realised effect, and that the true residual is likely to be larger. It also separates the amount of the bill above the ceiling, which is what we compute, from the amount patients would finally have paid after any other financing, which we cannot observe. Table S7 lists the assumptions.

**Change made: **Discussion, fifth paragraph; Table S7.

***7. Causal language and policy conclusions.*** *Statements such as “the financial fate of a trauma patient … was decided by a single factor” and “the binding failure … was the absence of cover, not its design” are stronger than the study design supports …*

 Thank you; we accept this. Both statements have been removed, along with others in the same register: “isolating the effect of the payment mechanism”, “removes that confounding by design”, “ruled out by the data themselves” and “for identical care at identical prices”. The abstract now states that the contrast is an association rather than a causal effect. The sentence on on-site eligibility verification is now framed as a hypothesis for future research, and the text notes that eligibility was not assessed among the uninsured. The Discussion separates what was observed from what is inferred, and the policy implications are stated as steps to test rather than as demonstrated effects.

**Change made: **Title; Abstract, Conclusions; Introduction, final paragraph; Discussion, first and second paragraphs; Conclusions.

***8. Study population and potential selection bias.*** *Please discuss how these exclusions may affect the payer distribution and observed financial burden … The title and conclusions should consistently refer to admitted patients or the study cohort.*

 We agree. Methods now states that patients treated in the emergency department without admission were outside the eligibility criterion, alongside those who died before admission. Methods and a redrawn Figure 1 also separate the two exclusion steps more clearly: 102 admissions financed through other schemes fell outside the study’s scope, leaving 1,609, and 10 privately insured admissions were then excluded for incomplete billing, leaving 1,599. A new limitation sets out what the exclusions mean: the payer mix describes admitted patients financed through the three study modes, not all crash victims. It also discusses the direction of the resulting bias, which is uncertain. Deaths before admission remove the most severe injuries from every group, while patients unable to reach or afford a tertiary centre are likely to be poorer and uninsured, so the burden on the uninsured may be understated. The title, abstract and conclusions now refer to road traffic accident admissions.

**Change made: **Title; Methods, Participants and eligibility; Figure 1 (redrawn); Strengths and limitations (sixth limitation); Conclusions.

**Minor comments**

***Study design and reporting.***

 Thank you. We have kept the STROBE checklist for cross-sectional studies, which we think fits best: each admission is observed once, and payer status, charges and OOPE are all measured from the same billing episode, with no follow-up after discharge. The abstract and Methods now describe the study as a retrospective, cross-sectional analysis, and Methods states that the unit of analysis is the admission. The checklist has been updated to Version 2 and now points to section headings rather than page numbers.

***Confidence intervals.***

 Done. The main medians (charges and OOPE by payer group, and OOPE by type of private payer) now carry bias-corrected and accelerated bootstrap intervals (5,000 resamples, with the admission as the resampling unit and a fixed random seed), and proportions carry Wilson intervals; Methods describes both. Because the intervals were re-estimated with the deposited script, some bootstrap limits differ slightly from Version 1. For example, the interval for the AB-PMJAY median charge is now ₹77,148–₹109,334, against ₹79,977–₹109,048 in Version 1. No point estimate changed.

***Interpretation of zero OOPE.***

 Agreed; “zero recorded hospital OOPE” is now used throughout, with a 95% CI for the AB-PMJAY proportion (94.3–100%).

***Terminology.***

 Thank you. Methods now defines the uninsured group as patients who registered no cover with the hospital for the admission. The Discussion describes them as admitted “with no cover registered with the hospital”, which does not exclude other forms of assistance.

***Road-user classification.***

 Agreed. The limitation now states that we draw no detailed conclusions from this variable.

***Presentation of results.***

 Thank you for the suggestion. New Table 5 gives, for each payer group, the median recorded OOPE, the proportion with zero OOPE and the proportion above each income threshold, all with denominators and 95% confidence intervals. The legend of Table 3 now distinguishes the mean from the median percentage.

***Reproducibility.***

 Thank you. The analysis script (RTA_OOPE_analysis.py), which reproduces every estimate in the article from the deposited patient-level file, has been added to the figshare deposit (version 3). Checking the supplementary file against the dataset turned up three errors, which we have corrected. The 25th, 75th and 95th percentiles in Table S6 did not match the deposited data (10.1, 31.7 and 84.1 are now 10.2, 31.9 and 84.2); the 90th percentile and median cited in the text were correct. The Notes sheet still said that patient-level data could not be shared, a leftover from an earlier draft that contradicted the deposit. The STROBE checklist gave an outdated count for the other or unspecified road-user category (437) instead of the 579 reported in the article. Rechecking the article itself, we found that the Fisher–Freeman–Halton p-value for road-user category was wrong in Version 1 (0.007); the correct value is 0.003, and it is corrected in Results. The conclusion it supports, that road-user category differed across payer groups, is unchanged.

 We are grateful for the time and care you gave to the manuscript.

 Warm regards,

 Rajesh Kamath
